# Spatiotemporal shaping of terahertz radiation produced by a two-color ultrashort flying focus

**DOI:** 10.1038/s41467-026-76228-6

**Published:** 2026-07-29

**Authors:** J. J. Pigeon, H. S. Markland, R. Boni, K. G. Miller, A. L. Elliott, J. Kendrick, M. Lim Pac Chong, P. Estrela, D. J. Gitlin, D. N. Purschke, J. P. Palastro, D. H. Froula

**Affiliations:** 1 Laboratory for Laser Energetics, University of Rochester, Rochester, NY USA; 2 Physics Department, University of Rochester, Rochester, NY USA; 3 Institute of Optics, University of Rochester, Rochester, NY USA

**Keywords:** Nonlinear optics, Terahertz optics

## Abstract

The flying focus features an intensity peak that propagates at a focal velocity that is decoupled from the group velocity of the medium. Control over the velocity of the focal point is predicted to revolutionize secondary source experiments by allowing velocity matching of physical processes at the core of nonlinear optics and high-field physics. Despite this promise, experimental demonstrations have lagged pioneering theoretical work due to the difficulty of creating an ultrashort flying focus in the laboratory. In this article, the spatiotemporal shaping of a terahertz pulse generated via gas-phase photoionization by a two-color, ultrashort flying focus is described. The combination of radial group delay provided by reflective, radially-stepped echelons and spherical aberration produced by an axiparabola generates a flying focus with sub- and superluminal focal point velocities. The flying focus efficiently generates terahertz radiation that exhibits fundamentally different spatiotemporal properties based on its velocity. We present detailed measurements of the spatial and temporal properties of the terahertz radiation and measure its focal velocity. This demonstration shows the utility of terahertz generation with a flying focus and enables future experiments in nonlinear optics with spatiotemporal control.

## Abstract (web summary)

Authors demonstrate spatiotemporal shaping of terahertz pulses generated through gas-phase photoionization using a two-color ultrashort flying focus. The approach enables tunable spatial and temporal pulse characteristics, offering a versatile platform for nonlinear optics and advanced terahertz source engineering.

## Introduction

Over the last two decades, substantial research has been dedicated to understanding the correlations between the temporal and spatial degrees of freedom of a laser pulse referred to as spatiotemporal couplings^1–23^. This work has been largely motivated by the potential of *spatiotemporal control* or the engineering of spatiotemporal couplings to advantageously impact the result of an experiment^3,5–10,14–18,20,21^. The flying focus, in particular, utilizes longitudinal chromatism or radial group delay to produce a focal point that propagates at a controlled focal velocity over many Rayleigh lengths^2,10^. The focal velocity may differ from the group velocity of the medium to affect physical processes by velocity matching.

Initial flying-focus demonstrations used a highly chromatic diffractive lens and a chirped pulse to produce an engineered focal velocity, albeit with a picosecond-scale pulse duration^2,3,21,22^. Despite this limitation, the chromatic flying focus was successfully applied to produce homogenous plasma channels^22,23^, seed an x-ray laser^13^, and steer the THz radiation produced in a single-color laser filament^9^. Although the latter demonstration was limited by a low (~10^−9^) THz power conversion efficiency, the researchers demonstrated steering of a pJ-class THz pulse, which illustrated the potential utility of the flying focus for THz production. Recently, the bandwidth limitation inherent in the chromatic flying focus has been addressed by replacing the chromatic aberration with geometric aberration to produce an ultrashort flying focus with <100 fs pulse duration^10^. The ultrashort flying focus has been considered for impactful applications that require femtosecond-class pulses or high intensity, including the production of TeV-class electrons in a dephasingless laser wakefield accelerator^5,6^ and the efficient generation and spatiotemporal shaping of terahertz (THz) radiation produced by two-color photoionization in gases^7,8^.

Two-color photoionization in gases^24–46^ offers a compelling route to the generation of intense THz radiation since the radiation generated in this scheme has a shorter pulse length (~ 0.1–1 ps) and can be focused more tightly than that produced using optical rectification or a photoconductive switch. Under conventional focusing, however, THz radiation constructively interferes in a Cherenkov-like cone with an angle ϕ determined by the velocity of the ionization front *v*_f_ and the phase velocity of the THz ***v***_THz_^44,45^:

1 $$\phi={\cos }^{-1}\left(\frac{{{{\mathcal{v}}}}_{{{\rm{THz}}}}}{{{{\mathcal{v}}}}_{f}}\right).$$

The velocity of the ionization front is faster than the THz phase velocity due to external focusing increasing the intensity resulting in photoionization occurring earlier in the two-color pulse as it comes to focus. More generally, the emission angle depends on the beam geometry, linear dispersion, and nonlinear propagation of the two-color pulse, making it difficult to predict or control in an experiment where parameters such as gas pressure are varied to optimize THz conversion efficiency. Moreover, in the filamentary propagation that typically accompanies THz generation, cycles of focusing and defocusing can cause THz to be emitted in a range of Cherenkov-like emission angles, resulting in a larger effective source size that limits how tightly the THz can be focused for applications.

Photoionization by a two-color flying-focus pulse can control the velocity of the photoionization front^22,23^ and has been predicted to alter the spatiotemporal properties of the emitted THz radiation^7,8^. Adjusting the flying-focus velocity above or below the THz phase velocity (*v*_THz_ ~ c) profoundly changes the THz emission process. A subluminal-flying-focus velocity (*v*_f ≤_ *v*_THz_ ~ c) creates a single-cycle THz pulse that interferes constructively on-axis, while a superluminal velocity (*v*_f ≥_ *v*_THz_ ~ c) produces a higher frequency THz pulse that constructively interferes off-axis to produce a well-defined emission angle resembling a Cherenkov shock. Regardless of the focal velocity, the flying focus creates a highly focusable THz emission as compared to THz produced without spatiotemporal control (i.e., a conventional Gaussian pulse as described in “Methods”). Therefore, the flying focus velocity can be used to control the emission angle, focal spot, and spectrum of THz radiation^7,8^. The use of a flying focus may also enable scaling the power of the laser beyond limitations related to ionization-induced refraction and multiple filamentation^37–41^ by spreading the laser intensity through the extended focus.

**Fig. 1:**
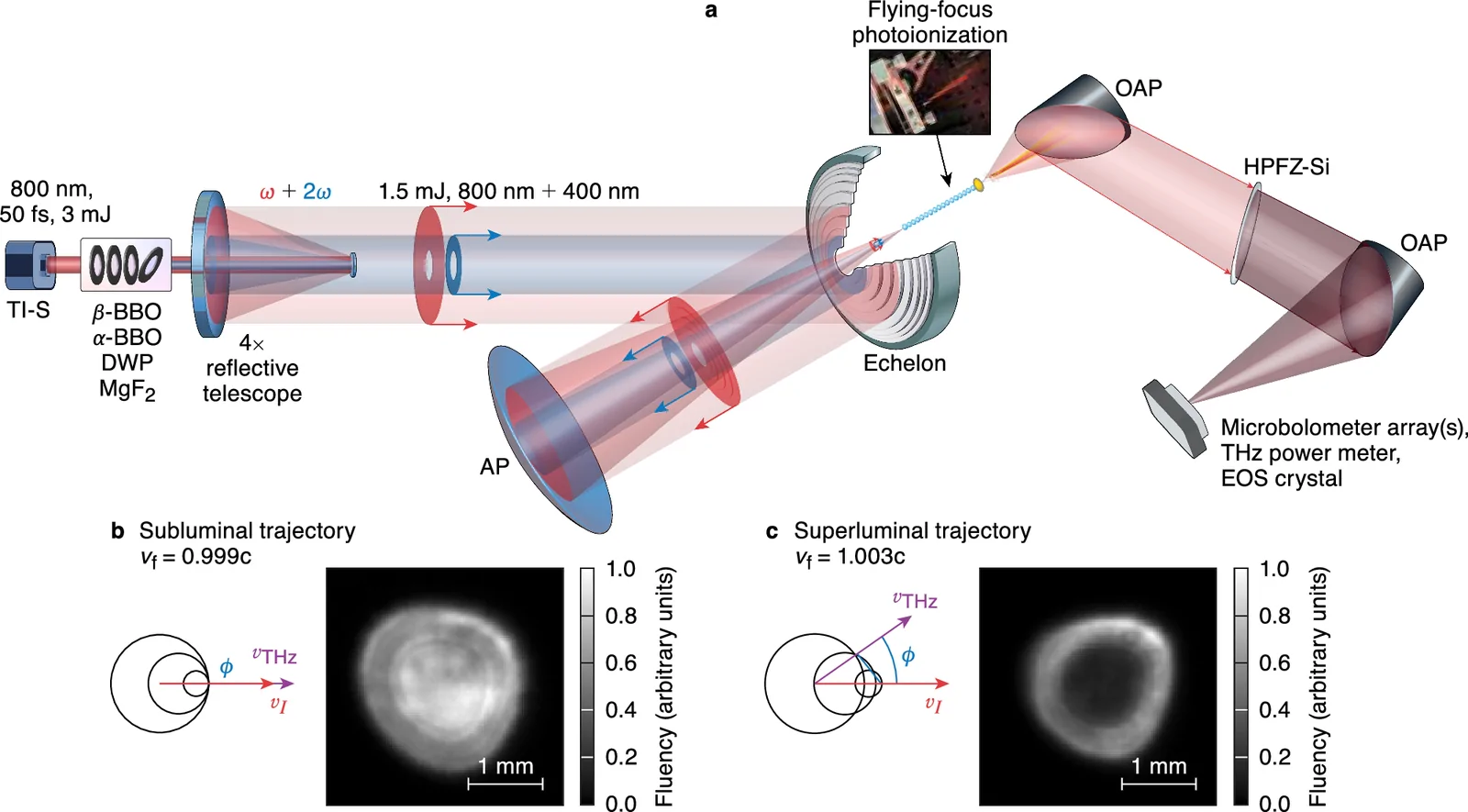
Terahertz production via photoionization by a two-color flying focus. Experimental set-up. **a** Schematic representation of the experiment, including formation of a two-color ultrafast flying focus using an axiparabola (AP) and an echelon pair. THz wavefronts and beam profiles. Visualization of the emitted wavefronts and experimental THz beam profiles generated by flying-focus pulses with (**b**) subluminal and **c** superluminal velocities. The beam profiles were measured 5 mm away from the focus and are representative of the shape of the collimated beam. DWP is a dichroic waveplate; OAP is an off-axis parabolic mirror; EOS is electro-optic sampling; HPFZ-Si is a high-purity float-zone silicon window; TI-S is a Ti: Sapphire laser; BBO is BaB_2_O_4_; ω and 2ω denote the fundamental and second-harmonic laser pulse.

In this article, we describe the efficient (10^–4^ power conversion efficiency in air) production and spatiotemporal shaping of THz radiation via two-color photoionization by an ultrashort flying focus. The flying focus was produced using a combination of a reflective, radially stepped echelon and an axiparabola^10^ that enabled a two-color ultrashort pulse to be propagated over nearly a centimeter at a constant sub- (0.999c) or superluminal (1.003c) velocity (Fig. 1). The radially-dependent focal length of the axiparabola^15,16^ focuses different annuli of the beam to different longitudinal locations in the extended focus, while the echelon adjusts the relative timing of the annuli as they come to focus to create a prescribed focal velocity over many Rayleigh lengths^10^. The echelons are fabricated with half-wavelength steps to impart radial group delay without changing the collimation of the beam. As a result, a single echelon can be applied to produce a two-color flying focus composed of a fundamental and its second harmonic. The two-color flying focus efficiently produced THz in air, enabled the control over both the spatial and temporal properties of the emitted radiation, and produced highly focusable radiation as compared to conventional focusing geometry. This capability to shape the spatial and temporal properties of THz radiation will enable advances in time-domain spectroscopy, free-space communication, and the production of high-intensity, long-wavelength fields for nonlinear optics and high-field physics experiments.

## Results

Figure 1a depicts the formation of a two-color flying-focus pulse using an f/5 axiparabola and an echelon pair. Part of the initial 3-mJ, 50-fs, 800-nm laser pulse was frequency doubled, and a birefringent time delay was used to ensure temporal overlap for the fundamental and second harmonic over the centimeter-long focal trajectory. Finally, a dichroic waveplate provided parallel polarization between the harmonics and the relative phase was controlled by rotating a 1-mm thick MgF_2_ plate that enabled THz power conversion efficiencies on the order of 10^–4^ in air. The emitted THz radiation was re-collimated by an f/3 off-axis parabolic mirror (OAP) before being focused by a second f/2 OAP. The spatial and temporal properties of the emitted THz were measured about the focus of the second OAP using microbolometer arrays and electro-optic sampling, respectively (see “Methods”). For spatial measurements, a low-frequency (LF) and high-frequency (HF) microbolometer array was employed, optimized for 0.4–4 and 20–40 THz. Although optimized for higher frequencies, the HF microbolometer was operated with a lock-in imaging scheme (see “Methods”) that enabled background-free measurements of frequencies <20 THz where the HF array has reduced sensitivity^42^. The transmission losses of the telescope and slight temporal broadening from the flying focus optics resulted in an approximately 1.5-mJ, 56-fs FWHM laser pulse in the focal range of the axiparabola (see “Methods”). For comparison, THz was also produced using a 3.0-mJ, 50-fs FWHM pulse with a conventional focusing geometry that produced a comparable filament length as the flying focus (see “Methods”). The conventional geometry, subluminal flying focus, and superluminal flying focus produced THz conversion efficiencies of 0.84 × 10^–4^, 0.86 × 10^–4^, and 1.2 × 10^–4^, respectively.

**Fig. 2:**
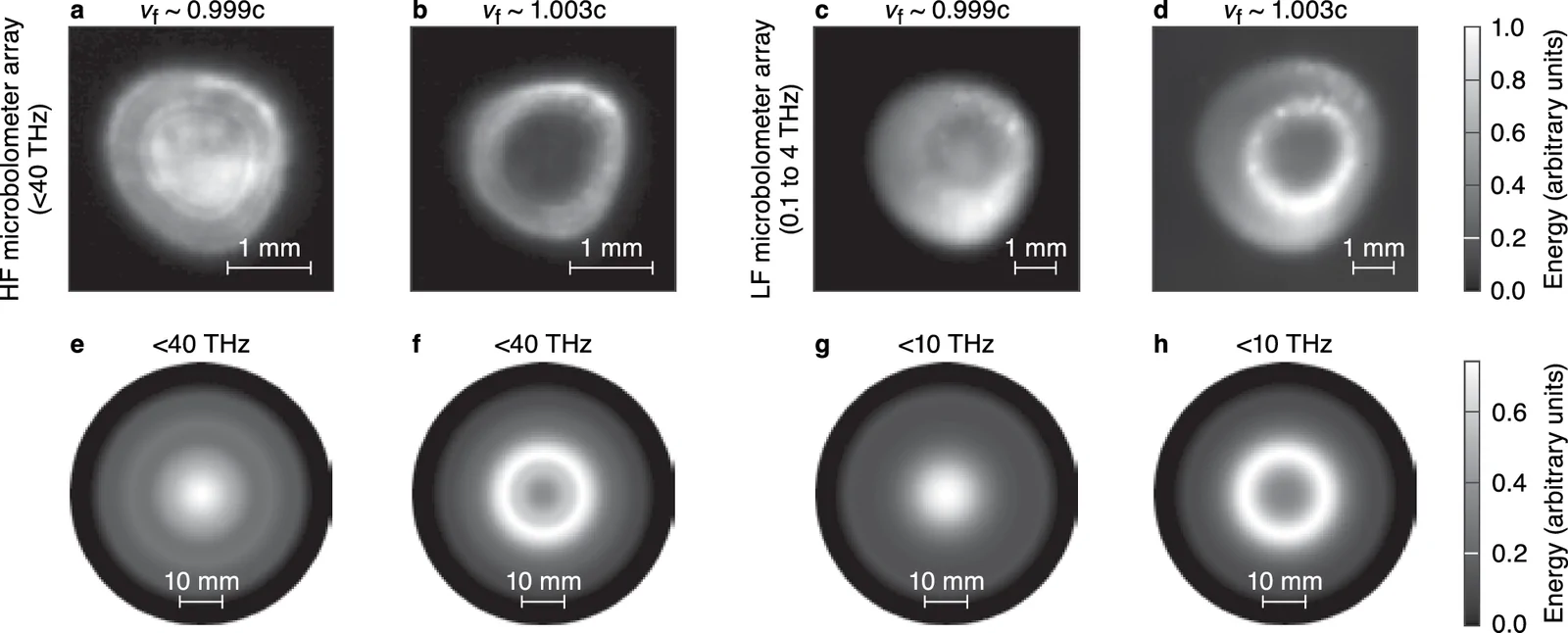
Subluminal and superluminal two-color flying-focus pulses produce on-axis and conical emission. Experimental THz beam profiles. THz far-field profiles produced with subluminal (**a, c**) and superluminal (**b, d**) flying-focus trajectories, measured with a high frequency (HF) microbolometer array (**a, b**) and low-frequency (LF) microbolometer array (**c, d**). Simulated THz beam profiles. **e–h** THz emission profiles from simulations initialized with corresponding focal velocity and frequency range of the experimental measurements directly above each panel. Note that the experimental beam profiles were measured 5 mm away from the OAP focus and that the simulations were measured 100 mm from the filament. The spatial sizes were in agreement after accounting for the differences in magnification.

Varying the focal velocity of the flying focus produced fundamentally different spatial beam profiles. Figure 2 displays measured and simulated spatial beam profiles of the THz radiation in both the low- and high-frequency ranges. The subluminal velocity generated THz that was peaked on-axis, while the superluminal velocity produced a ring-shaped beam consistent with the Cherenkov effect. The simulations reproduce the qualitative shape, size, and divergence of the beam after accounting for the differences in magnification, but agreement was better with the HF spatial measurements than the LF measurements. This may be due to the simulations not capturing frequencies <1 THz (see “Methods”). Other discrepancies are mainly related to experimental realities, including aberrations in the optics, details of the initial pulse profile and spatio-spectral phase, as well as the introduction of a tri-foil shadow due to the up-collimating telescope (see “Methods”). The simulations also did not account for absorption and dispersion of IR-active minor air constituents such as H_2_O vapor.

**Fig. 3:**
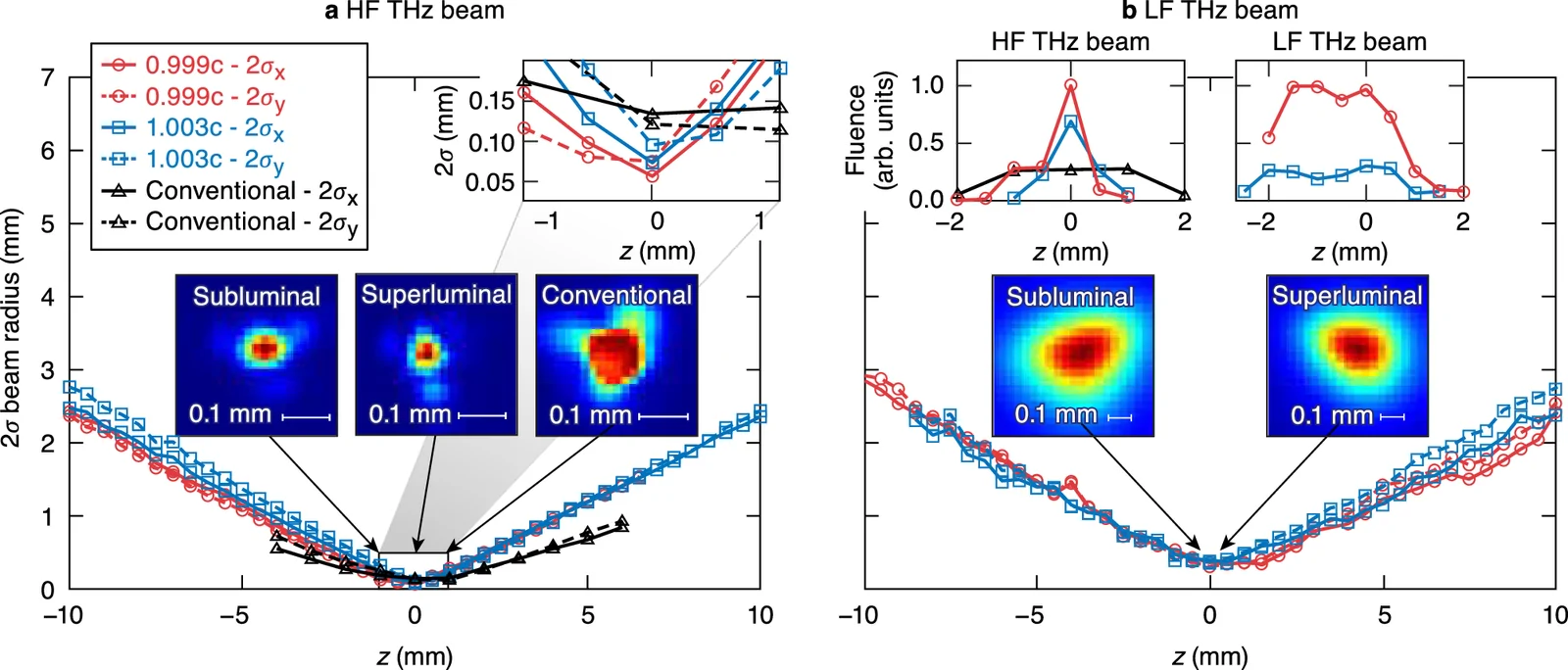
Two-color flying-focus pulses generate more tightly focused THz radiation. Spatial beam profiles measurements about the focus. The RMS beam radius (2σ_x_, 2σ_y_) and spatial beam profiles near the focus of the THz beam are measured with the HF microbolometer array (**a**) and LF microbolometer array (**b**). The inset plot of (**a**) shows the beam radius in the vicinity of best focus, while the inset plots of (**b**) show the fluence measured with the HF and LF microbolometer array. The inset images in (**a**) and **b** are beam profiles measured near the position of best focus.

Figure 3 demonstrates that the flying focus produced high-frequency THz radiation that was more focusable than that produced with a conventional focusing geometry. To directly compare the ring-shaped and on-axis beam shapes, the RMS beam width (σ_x_, σ_y_) was determined by calculating the second moment of the fluence with respect to two directions (x, y) measured relative to the beam centroid^46^. Figure 3a, b show the 2σ beam radii measured about the focus with the HF and LF microbolometer arrays, respectively. The sub- and superluminal flying focus produced THz focused to a minimum beam radius of 57 µm and 95 µm, respectively, while the THz generated by conventional focusing had a minimum radius of 120 µm (Fig. 3a). Measurements of the improved focusing of the flying-focus-produced THz are consistent with previously simulated THz virtual source sizes^7^ and can be understood by considering the angle of emission (see Eq. 1). As opposed to the conventional geometry that produces a range of Cherenkov-like angles due to nonlinear propagation, the flying focus produces THz at a well-defined angle due to the constant ionization front velocity, resulting in improved focusing. For the low-frequency measurements (0.4–4 THz), both flying-focus velocities produced THz that focused to a minimum spot size of ~ 360 µm, which corresponds to the diffraction limit. The peak fluence of the THz radiation was also determined from these measurements [see inset of Fig. 3b], which shows that the flying focus produces higher fluence HF radiation than the conventional focusing geometry. Moreover, measurements of the LF THz beam show an increased fluence obtained with the subluminal velocity, indicating that this velocity produced more LF radiation than the superluminal case.

**Fig. 4:**
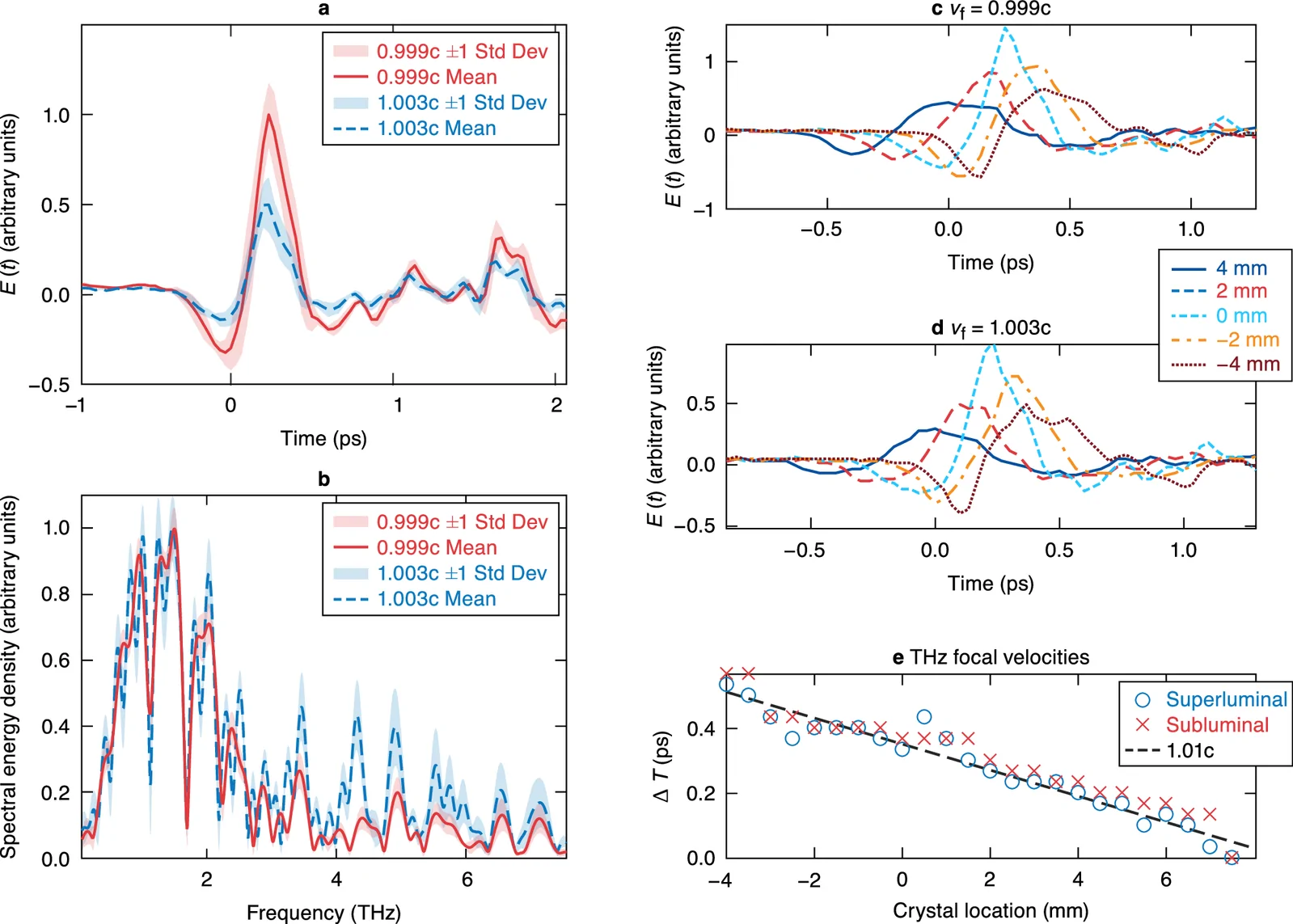
The flying focus focal velocity tunes the THz spectrum without altering the THz focal velocity. Electro-optic sampling measurements of the THz electric field and spectrum. **a** The mean THz electric field and standard deviation from 5 measurements performed using electro-optic sampling near the THz focus at z = 0 (see Methods) for the sub- and superluminal flying-focus velocities. **b** The Fourier transform of the mean +/– one standard deviation of the data presented in (**a**). Determination of the THz focal velocity. Time-domain measurements of THz produced with the subluminal (**c**) and superluminal (**d**) flying-focus pulses, measured about the focus of the second OAP in Fig. 1. **e** Measurements of the focal velocity of the THz pulse obtained using electro-optic sampling by translating the crystal through the THz focus. ∆T is the relative time between the peak THz electric field and the probe pulse.

Similar to the spatial data presented above, time-domain measurements were performed about the focus of the OAP to map the emitted field and to measure the focal velocity of the THz produced using the flying focus. Figure 4a shows the THz electric field measurements for the sub- and superluminal flying focus velocities, and Fig. 4b depicts their Fourier transforms. The electric field of the THz produced with the subluminal flying focus was measured to be 2× larger than the superluminal case. This observation was consistent with the increased fluence at low frequencies determined by the LF microbolometer array (see inset of Fig. 4b) since the electro-optic sampling method was only sensitive to frequencies below 7 THz. Regardless of longitudinal position about the focus, the superluminal flying-focus produced higher frequency THz radiation than the subluminal velocity (Fig. 4b). To illustrate this, both the time- and frequency-domains are presented as the mean and standard deviation of 5 measurements taken near the THz focus (z = 0). With the subluminal flying focus, the ionization front lags the THz pulse and generates new radiation behind that generated previously, thereby lengthening the pulse in the time domain and lowering the THz frequency. The ionization front driven by the superluminal flying focus, however, creates new THz radiation ahead of the previously generated radiation that interferes in a Cherenkov-like shock that shortens the THz pulse duration, generating higher frequencies than the subluminal case. The modulations present in Fig. 4b are likely due to H_2_O absorption in the laboratory air and temporal reflections in the electro-optic crystal. Supplementary Fig. S2 shows this data after time-domain filtration to remove the modulations behind the main THz pulse, along with electro-optic sampling data obtained using the conventional focusing geometry.

To determine the focal velocity of the THz, a series of electro-optic sampling measurements for the sub- and superluminal flying-focus were obtained by translating the electro-optic crystal through the THz focal region and observing the time at which the THz and near-IR probe were synchronized. These measurements show that the peak THz field moves in time relative to the group velocity of the probe pulse (∆T) and that this time depends on the focal location (Fig. 4c, d). When plotting these measurements as a function of the crystal location, the THz radiation was found to propagate with a focal velocity near 1.01c, regardless of the flying-focus velocity used to produce the THz (Fig. 4e). These results agree with previous measurements of the focal velocity of a THz pulse produced using conventional two-color photoionization, suggesting that using the flying focus to tune the properties of the THz radiation does not alter the THz focal velocity—a change that would deleteriously affect applications^47^.

## Discussion

The spatiotemporal measurements presented above show that the flying focus can be used to modify and even improve the transverse spatial distribution and frequency content of an emitted THz pulse. Photoionization driven by a flying focus resulted in efficient (10^–4^) THz production, spectral tunability, and near-diffraction- limited focusability, underscoring the potential utility of this technique. Further optimization of the focal velocity or f/# of the axiparabola could result in greater efficiency. This is especially true for more powerful pump lasers, since the extended focus can mitigate saturation mechanisms by lowering the local intensity. The spatial and spectral measurements were in good agreement with the results of idealized, radially-symmetric simulations presented here and previously^7^, despite imperfections in the experimental system related to slight misalignment and the initial radial group delay of the input laser pulse. Note that the superluminal flying focus also resulted in slightly higher (1.2 × 10^–4^) conversion efficiency as compared to the subluminal case (0.86 × 10^–4^). A similar trend was previously reported in ref. ^9^ and predicted by simulations^7^.

This technique provides control over the spatial and temporal properties of THz radiation but has important limitations. Most notably, precise alignment of the echelon and axiparabola was required to produce the correct focal velocity of the flying focus. With increasing misalignment, the THz profile began to resemble the profiles produced using the axiparabola alone, as the echelon was no longer properly structuring the radial group delay of the pulse. In addition, spatiotemporal structuring with large aperture optics requires a reflective telescope and multi-meter propagation that necessitates a time-delay compensator for nonlinear frequency conversion with a collimated beam. Finally, the use of an all-reflective telescope with center obstruction resulted in a 50% reduction in the pulse energy (see “Methods”). Future work will include extending the flying focus optic fabrication technique to integrate the echelon and axiparabola on a single optic, simplifying its application. Furthermore, recently developed methods of producing a tunable ultrashort flying focus using adaptive optics may prove useful for optimizing THz generation and provide rapid tuning of the THz beam profile and frequency content^48^.

The spatiotemporal shaping of THz produced via gas-phase photoionization by a two-color ultrashort flying-focus pulse was demonstrated. The ultrashort flying focus was created with an axiparabola and echelon pair capable of structuring the radial group delay of a fundamental and second-harmonic pulse with a single echelon. The flying focus produces competitive THz conversion efficiencies in air compared to state-of-the-art experiments, but with improved focusing capability and spectral control, suggesting that this source could be relevant to high-field physics applications after optimization. The flying-focus optics are easily extendable to other laser wavelengths and up to relativistic intensities, enabling further experiments in nonlinear optics and high-field physics with spatiotemporal control.

## Methods

### Laser and optical system

Ultrashort flying-focus pulses were produced using a 3-mJ, 1-kHz, 50-fs, 800-nm laser pulses from a commercial Ti: Sapphire laser system (Coherent Astrela). Laser pulse durations were determined using a commercial FROG device (Swamp Optics) prior to up-collimation and spatiotemporal structuring with low-GDD optics. The initial diameter of the laser beam was 15mm (1/e^2^ diameter) and was collimated to 60 mm by a custom, all-reflective telescope featuring an aspheric convex primary mirror (30-mm diameter) and a concave spherical secondary mirror (545-mm radius of curvature, 100-mm diameter). The on-axis design resulted in the production of an up-collimated beam with a central obstruction, resulting in ~50% throughput. As a result, 1.5-mJ pulses were used to drive photoionization and THz production.

### Production of an ultrashort flying focus

The flying focus was produced using a combination of all reflective radially stepped echelons and an axiparabola^10^. The echelon shapes were designed based on an analytic model that is described in detail elsewhere^18^ and provides a relationship between the radial group delay imparted by the echelon τ_D_, the desired focal velocity *v*_*f*_, and a radially dependent focal length *f*(r):

2 $$c\frac{d{\tau }_{D}}{{dr}}=\left[1-\frac{{v}_{f}}{c}+\frac{{r}^{2}}{2{f}^{2}\left(r\right)}\right]\frac{{df}(r)}{{dr}},$$

where for an axiparabola

3 $$f\left(r\right)={f}_{0}+L{\left(\frac{r}{R}\right)}^{2}.$$

The pulse was focused by an f/5 (f_0_ = 500 mm) axiparabola with an extended focus of L = 10 mm and a clear aperture radius of R = 50 mm. The radially stepped echelons were designed based on the above formulas to produce nominally *v*_f_ = 0.999c and *v*_f_ = 1.003c focal trajectories, which were validated using spectral interferometry^10^. The spectral interferometric measurements were also used to measure the impulse frequency response of the echelon and axiparabola system, which was found to be approximately 26 fs FWHM^10^. Using this impulse frequency response, we estimate the pulse duration in this experiment to be 56 fs FWHM in the focal range of the axiparabola. The echelons were fabricated using a fused silica deposition technique with half-wavelength steps (400 nm), which ensured the radial group delay structuring did not introduce unwanted phase front curvature. The axiparabola was produced by applying the same fused silica coating technique on an f/5 spherical mirror to produce the desired axiparabolic shape. Both the echelon and axiparabola received a silver coating that minimizes the GDD for both 800- and 400-nm light. Note that the echelon and the axiparabola were underfilled by using a 60-mm-diameter beam due to experimental constraints. As a result, the extended focus in the experiment was reduced to 6 mm, but the trajectories remained identical as previously studied using spectral interferometry and numerical simulations^10^. The peak intensity achieved in this experiment was reduced from that produced by an f/5 focusing geometry due to the extended focus of the axiparabola. The peak intensity can be estimated by calculating the peak intensity and reducing it by the ratio of the Rayleigh length to the extended focus, which is ~1/237 for the experimental conditions. As a result, we estimate that a peak intensity of ~5.5 × 10^14^ W/cm^2^. Interferometric measurements of the echelon profiles used in the experiment are presented in Supplementary Fig. S1.

### Production of a two-color flying-focus with time-delay compensation

For efficient THz production with the flying focus, it was critical to perform second harmonic generation before up collimation and spatiotemporal structuring. However, the relatively long (4 m) propagation distance in air that was necessary to perform the optical manipulation required delay compensation of the SHG pulse. To accomplish this, a 25-mm-diameter, 100-µm-thick β-BBO was used to frequency double 2.7% of the incident fundamental laser light. After frequency conversion, a 5.8-mm-thick, 40-mm-diameter, *α*-BBO crystal cut at a crystal angle of 60° was employed to delay the fundamental light relative to the second harmonic. After the time-delay compensator, a 45-µm-thick, 30-mm-diameter, dichroic waveplate ensured parallel polarization between the first- and second-harmonic pulses. Finally, a 1-mm-thick uncoated MgF_2_ plate was used at a 14° angle of incidence to finely control the relative GDD and obtain efficient THz production. Note that the MgF_2_ plate was rotated to ensure that the relative delay was optimized.

### THz spatial beam profile measurements

Terahertz spatial beam profile measurements were performed at 1-kHz repetition rate using two separate VO_x_ microbolometer arrays optimized for either low- or high-frequency THz radiation (HF or LF). The LF microbolometer array (Boston Electronics MICROXCAM-384-i-THz) had a 13.44 mm ×10.08 mm active area with a 35-µm pixel pitch. The LF camera preferentially detects 0.4–4 THz and features a thick high-purity-float-zone-Si window. The HF camera (Teledyne FLIR Tau2 640) was 10.9 mm × 8.7 mm with a 17-µm pitch. The HF camera was optimized to detect 20–40 THz and therefore required an every-other-frame background subtraction, which was implemented similarly to that presented in ref. ^42^. Several HPFZ-Si windows were placed in the collimated space of the THz beam and were used to prevent saturation near the focus. The THz radiation was re-collimated by an f/3 (228.6 mm focal length) off-axis parabolic mirror (OAP) before being focused by a second f/2 (101.6 mm focal length) OAP.

### THz electro-optic sampling (EOS) measurements

A small reflection from the main beam was separated before the frequency-conversion stage to provide a probe beam for EOS measurements. The probe was transmitted through a hole in the focusing OAP and combined with the focused THz in a 100-µm-thick GaP crystal. After being rotated by the THz wave, the probe polarization was analyzed using a Wollaston prism, and the depolarization was measured using a commercial balanced photodiode (Newport Nirvana) with lock-in amplification. To map the temporal profile through the focus, the GaP crystal was translated in 0.5-mm steps, and sequential EOS measurements were performed. For measurements of the THz focal trajectory, the temporal location of the maximum EOS signal was plotted as a function of GaP crystal location, as performed in ref. ^46^.

### Two-color THz production with conventional focusing geometry

THz was generated via two-color photoionization using a conventional focusing geometry to compare the best focus with and without spatiotemporal control. An initially 15-mm (1/e^2^ diameter) beam was focused by a 500-mm-focal-length lens (f/33) to produce a filament of comparable length (6 mm) to that produced with the axiparabola and echelon configuration. A 100-µm-thick β-BBO and a 45-µm-thick dichroic waveplate were positioned in the focusing beam at a distance of 230 mm from the focal point and filament location. The THz beam produced in this configuration was then re-collimated and focused by identical OAPs as those used to manipulate THz produced with the flying focus. In addition to the spatial beam profile measurements presented in this article, the electric field of the THz produced with the conventional focusing geometry was also measured at its focus. Electro-optic sampling measurements under identical conditions were performed, and the resulting Fourier transform is presented in Supplementary Fig. S2. For comparison, this data is presented with that obtained with each flying focus velocity [data presented in Fig. 4b. In Fig. S2, a temporal filter was applied after the main THz pulse (for times greater than 1 ps) prior to performing the Fourier transform to reduce the spectral modulations attributed to H_2_O absorption in laboratory air and temporal reflections in the electro-optic crystal.

### Simulations of THz production

Simulations of THz generation were conducted using a radially symmetric formulation of the unidirectional pulse propagation equation (UPPE)^49–51^, implemented as described in ref. ^52^. The UPPE included nonlinear source terms accounting for the nonlinear polarization density of the gas, the current density of the plasma, and the energy loss due to photoionization. The total electric field, including both the two-color laser pulse and generated THz radiation, was computed at each discretized propagation step along *z* using a second-order predictor–corrector scheme. The electric field and plasma density were evaluated in both configuration (*r*, *t*) and spectral (*k*_*r*_, *ω*) space using quasi-discrete Hankel transforms and fast Fourier transforms, respectively. To accurately model propagation of the flying focus pulse as well as THz generation and propagation, a large computational domain was required. Even with this large domain, it was not possible to simulate THz frequencies less than 1 THz. The simulations used 8000 steps along *z*, 6144 grid points in *r*, and 24576 grid points in *t*, corresponding to a physical domain of 14 mm in *z*, 4.5 mm in *r*, and 5.4 ps in *t*. The far-field THz profiles were obtained by applying a single linear propagation step to the THz field at the final *z* step. All computations were performed with the JAX library for Python, which provides native support for GPU acceleration. Each simulation took approximately 5.2 h to complete on a single NVIDIA A100 GPU on the Perlmutter system at NERSC (account m4372). To maximize THz yield, multiple trial runs were employed to empirically determine the optimal phase between the fundamental and second-harmonic pulse.

## Supplementary information


Supplementary Information
Transparent Peer Review file


## Data Availability

The spatial beam profile and time-domain data underlying the results presented in this paper are publicly available at https://doi.org/10.60593/ur.d.32609970.
