# Supplementary material for: Spatiotemporal shaping of terahertz radiation produced by a two-color ultrashort flying focus
Source: Nat Commun. 2026 Jul 29;17:9173. doi: 10.1038/s41467-026-76228-6 (PMC13521897; doi:10.1038/s41467-026-76228-6)
Supplement: Supplementary file 1 [file 41467_2026_76228_MOESM1_ESM.pdf]

Supplementary information

Spatiotemporal shaping of terahertz radiation produced by a two-color ultrashort flying focus

J. J. Pigeon[1*], H. S. Markland[2], R. Boni[1], K. G. Miller[1], A. L. Elliott[2], J. Kendrick[1], M. Lim Pac Chong[1], P. Estrela[2], D. J. Gitlin[3], D. N. Purschke[1], J. P. Palastro[1,3], and D. H. Froula[1,2]

[1]*Laboratory for Laser Energetics, University of Rochester, Rochester, New York 14623, USA*
[2]*Physics Department, University of Rochester, Rochester, New York 14623, USA*
[3]*Institute of Optics, University of Rochester, Rochester, New York*
**jpigeon@ur.rochester.edu*

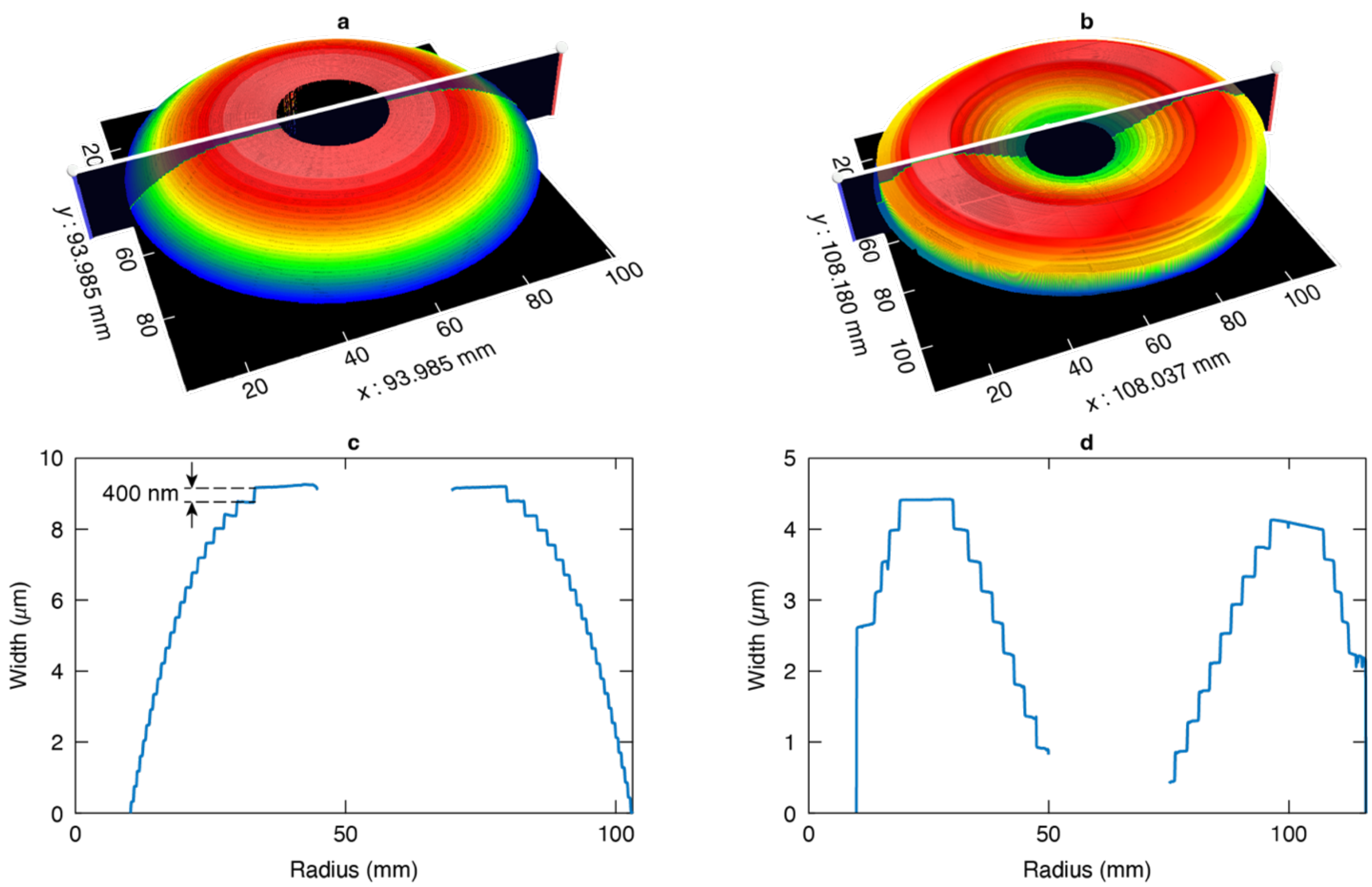

Figure S1. Surface profiles of the echelons used to produce the flying focus. (a, b) Interferometric measurements of the surface profiles of the echelons used to produce subluminal (a) and superluminal (b) flying-focus velocities with an f/5 axiparabola having an extended focal length of 10 mm. (c, d) Lineouts depicting the half-wavelength steps of the echelon for the subluminal (c) and superluminal (d) flying-focus velocities.

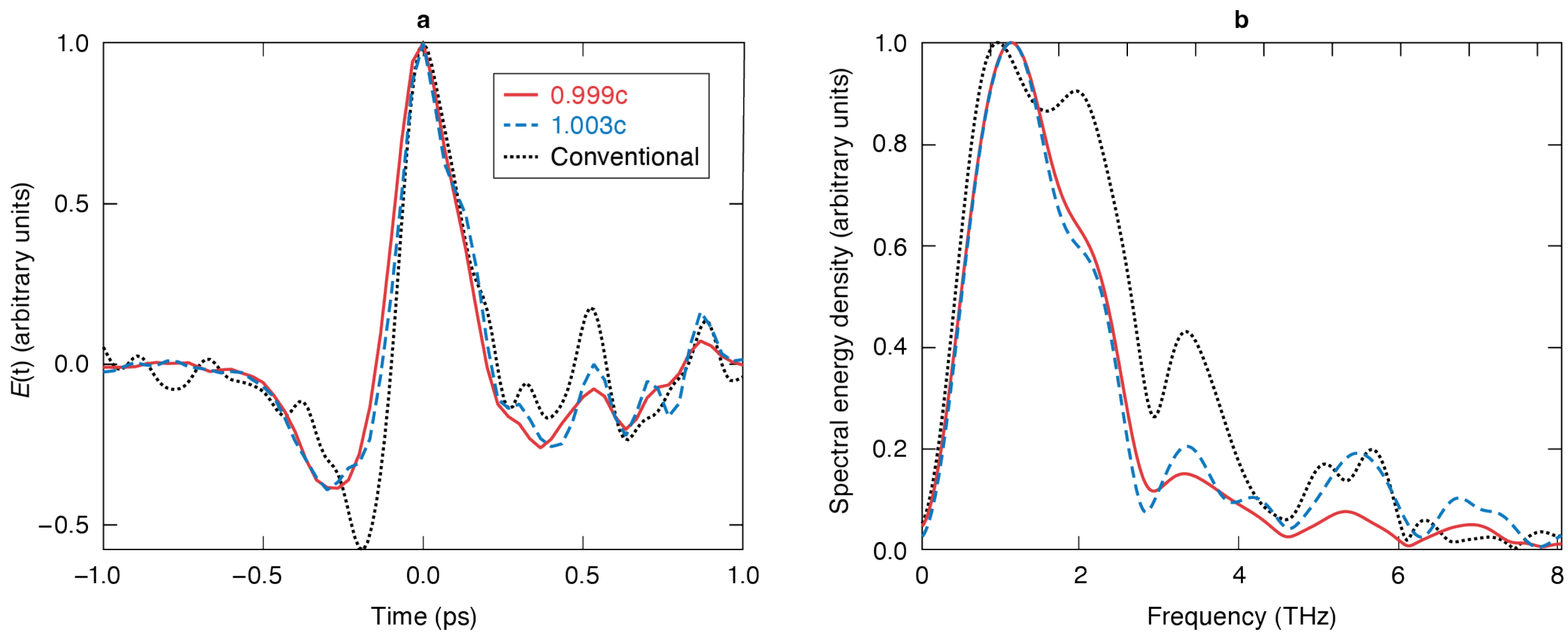

Figure S2. Filtered electo-optic sampling measurements (a) The normalized electric field of THz produced with the conventional focusing geometry and each flying-focus pulse both obtained via electro-optic sampling in 100-μm thick GaP crystal. (b) The associated spectra obtained via Fourier transformation after application of a temporal filter beyond 1 ps to reduce the spectral modulations present in Fig. 3(b).
