# Supplementary material for: Spatiotemporal shaping of terahertz radiation produced by a two-color ultrashort flying focus
Source: Nat Commun. 2026 Jul 29;17:9173. doi: 10.1038/s41467-026-76228-6 (PMC13521897; doi:10.1038/s41467-026-76228-6)
Supplement: Supplementary file 2 [file 41467_2026_76228_MOESM2_ESM.pdf]

nature portfolio

Peer Review File

Spatiotemporal shaping of terahertz radiation produced by a two-color ultrashort flying focus

Corresponding Author: Dr Jeremy Pigeon

This file contains all reviewer reports in order by version, followed by all author rebuttals in order by version.

Version 0:

Reviewer comments:

Reviewer #1

(Remarks to the Author)
The manuscript describes an experimental study investigating the shaping of terahertz radiation from a plasma generated in air using a two-color femtosecond laser pulse and the flying focus technique. A recently developed method for controlling the focus velocity of a laser pulse with a femtosecond duration was used to demonstrate that terahertz radiation can be efficiently generated in air through two-color laser-induced photocurrent. This ultrafast flying focus technique can be used to tune the THz radiation's spectrum and improve its focusability.
The proposed concept is original, and the experimental measurements are convincing and important for the future application of broadband terahertz pulses. However, some points need more detailed descriptions before publication.

Therefore, I believe the manuscript requires mandatory revisions before it can be accepted for publication in Nature Communications.

Questions to Address:
1. One significant advantage of the ultrashort flying focus technique is the ability to produce a flying focus with a velocity variation of 0.3%, all while keeping the pulse duration below 100 fs. Could you estimate or measure the effective pulse duration of your initial 50 fs laser pulse at the focal point of your parabola? The THz yield is sensitive to the initial pulse duration; therefore, this parameter is important for improving the THz conversion.
2. In the case of the superluminal ionization front, a conical-shaped THz radiation is observed, which is attributed to Cerenkov-like emission. A similar observation was made in reference [9], where the authors predicted an increase in THz energy for superluminal velocities compared to conventional focusing and a decrease in THz energy for subluminal velocities. Their velocities were much higher, but do your experiments or simulations show similar trends?
3. I am impressed by the section on characterizing the THz focusability. What is the proportion of THz energy in the spectral range above 10 THz? Based on the EO sampling measurement, it looks like this high frequency part is negligible and that a large part of the signal measured by the HF bolometer is actually at low frequency (unless you use a low pass filter).
4. Figure 2 shows that the numerical simulations qualitatively reproduce the shape of the measured THz profile, but the agreement is not very good. These simulations are very complex, but one important aspect is unclear in your description of two-color THz generation: The photocurrent created by the two-color pulse depends strongly on the phase difference between the 800-nm pulse and the 400-nm pulse. However, due to dispersion, these two pulses have different velocities in air, which induces phase slippage during propagation and eventually leads to destructive interference in the THz emission when the plasma source is too long. This is why most people limit the length of the plasma source with a two-color laser to a few millimeters. In your case, however, you produce a relatively long plasma source (6 mm), yet you do not discuss the problem of phase slippage between the 800- and 400-nm pulses. You only mention the ionization front velocity. However, to achieve optimal THz generation, it appears that you must independently control the 800-nm and 400-nm pulses to counteract the dispersion in air and plasma. Could you comment on this?
5. Figure 4 shows the THz temporal shape and the corresponding spectra measured with the two flying focus cases. Could you also compare these with the "conventional case"? Also, the measured signal appears quite noisy for such a high-energy THz pulse. Could you comment on the origin of the numerous absorption lines in Fig. 4b's spectrum? Are they due to the GaP crystal or humidity in the air?

Reviewer #2

(Remarks to the Author)
Key results:
The authors report on spatio-temporal shaping of single-cycle terahertz pulses produced by a flying focus via two-color photoionization in air. It is shown that the shape of the far-field THz beam profile can be tuned from the typical Cherenkov-like ring to a cleaner flat-top by adjusting the focal velocity of the flying focus. This method allows a tighter focusing of the THz beam compared to conventional two-color air-plasma THz generation. Superluminal focal velocities yield higher frequency content in the generated THz pulses. The experimental results are in good agreement with previous theoretical work of the group.

Reviewer report:
The interpretation of the provided data is valid and the authors' conclusions are comprehensible. The generated THz pulses were characterized spatially and temporally depending on the flying focus properties. However, a central claim of the work, the spectral/temporal control of the terahertz radiation is not well supported by the provided data, as only two different data sets (1 subluminal versus 1 superluminal) are shown. The results are certainly significant for the THz community developing and using THz sources based on two-color photoionization in gases, due to the enhanced spatial properties of the focused THz beam. Ultrafast flying optical and THz foci could also be used for novel application in nonlinear optics and high-field THz science and therefore be interesting to a broader audience.
In general, the quality of the data and presentation is appropriate. The content of Fig. 4e is not properly explained though. What is shown here? What is Delta T? The plot title is also wrong. The presented work is clearly focused on the experimental realization of previous theoretical work (Refs. 7,8). In this regard, the manuscript often fails to provide the relevant physical background to the reader. Without knowing the referenced theory work in depth, it is not clear why a flying focus should enable tighter THz beam focusing than conventional two-color photoionization (line 152). In addition, it is not clear why a superluminal flying-focus produces higher frequency content than the subluminal (line 171). Apart from these points, the manuscript is well accessible and compelling in its conciseness. Previous work seems to be considered thoroughly in the introductory part and is referenced appropriately.

Suggested improvements:
• As mentioned above, additional EOS data in Fig. 4 for more focal velocities would strengthen the work and the claim of spectral control.
• The agreement between experiment and simulation in Fig. 2 is debatable. There is no length scale provided in the simulated data. A more detailed discussion of agreements and deviations is desirable.
• The structure of the paper could be optimized to avoid repetitions. Some results are presented already before the beginning of the Results section (line 99 and subsequent).
• Minor language issues: doubling of words in l. 383, "MgF2 plate" (l. 408), "Wallaston prism" (l. 422), missing word? in l. 110, "mission" (l. 143)

Reviewer #3

(Remarks to the Author)
The authors experimental demonstrate the effect of a flying-focus (FF) to modify the phase matching for terahertz pulse generation in air with two-colour (w-2w) pulse excitation, using an approach similar to that proposed e.g. in Ref. 7, here using reflective optics to avoid group walkfoff between the w- and 2w-pulses.
The influence on the subsequent THz beam shape is convincing, and reasonably consistent with predictions of detailed spatiotemporal propagation simulations. This method is not only of interest to researchers in the field of THz pulse spectroscopy, but also serves as another example of using a FF geometry to tailor nonlinear optical generation processes.

However, there are several key points the author's should address before I could recommend publication of the manuscript.

1. As with any modified experimental scheme, requiring a more complex apparatus, one needs to clearly address any disadvantages that might arise, not only the apparent benefits over the "conventional" approach.
In the current manuscript, the only comparison of the FF and conventional method is in the beam radius vs distance about the subsequent focal plane (Fig. 3(a)). The author should include the beam profiles of the conventional method as well (especially at z=0), so the reader can compare with the FF cases.
Also, the pulse/spectrum for the conventional case should be given in Fig. 4(a,b), as well as a statement on the pulse energies/peak focal fluence in all three cases. If the FF approach suffers from some additional losses, these should be stated, but a comparison still made.

2. The authors should address better the true gain from the subluminal case compared to conventional. In the Introduction, the conventional conical emission is described only as a disadvantage ("impossible to control", "complicates collection … of the radiation"). In practice, the conventional cone angle is still very manageable, the beam is linearly polarized, and subsequent foci still have a clean on-axis maximum (the cone only leading to weak side lobes). Moreover, the "donut" collimated beam even has advantages in terms of allowing on-axis elements to filter the optical pump beams and reintroduce collinear optical beams for detection. If the authors can demonstrate that there is a true efficiency gain from the FF geometry, this needs to be stated (although I suspect this is not the case, as it is not addressed in the manuscript).

3. The apparent increase in spectral intensity (from ~3-7 THz) in Fig. 4(b) for the superluminal case is not convincing. Why are the spectra strongly modulated - due to reflections in the time-domain? The Fourier analysis should be improved,

including error margins for the high-frequency signal to ensure this section of the spectrum is reliable. As mentioned in point 1 above, the spectrum from the conventional method has to be included.

4. One important issue is that by spreading the focal region with a FF, the peak intensities reached are lower. Hence, plasma densities will be lower, which will be a penalty unless one reaches the saturation regime. The authors need to specify what conditions (i.e. pump opulse energies) the FF could maintain the THz generation efficiency.

5. The used definition of the beam radius is not rigorous. Here one should use the rms width calculated relative to the center of mass, and also the on-axis peak fluence. At present, one of the most important conclusions is based on this subjective metric.

6. The beam profiles in Fig. 1(b,c) are initially confusing, and only become clear in reaching Fig, 2. The authors should state where they are measured relative to the focal plane (i.e. sufficiently far from the focus that they represent the shape of the collimated beam, not the focal profile).

7. Some minor issues: The Methods states a 4x magnification of the optical pump beams, but it is given as 10x in Fig. 1(a)? For the conventional method, was the beam expander also used? “Wallastan” -> “Wollaston”.

Version 1:

Reviewer comments:

Reviewer #1

(Remarks to the Author)
The authors have satisfactorily addressed all of the referees' questions. I therefore believe that the manuscript deserves to be published in Nature Communications.

Reviewer #2

(Remarks to the Author)
All of my previous concerns have been appropriately addressed by the authors.
The revised manuscript now provides substantially more physical background on why control over the flying-focus velocity is beneficial for THz generation and how it influences the THz emission characteristics, focal spot size, and spectral content. These additions significantly improve the accessibility of the work for a broader audience.
The additional conversion-efficiency and THz-fluence measurements strengthen the authors’ claims and demonstrate a measurable, albeit moderate, benefit of the flying-focus approach compared to conventional excitation geometries. Overall, I believe that the revised manuscript is suitable for publication in Nature Communications.

Reviewer #3

(Remarks to the Author)
The authors have satisfactorily addressed the issues raised for the original manuscript. They state now clearly a net increase in focal peak field and conversion efficiency compared to the "conventional" geometry, despite the losses in optical pump pulse energy traversing the telescope/echelon/axiparabola. Hence this experimental approach could have real benefits for non-linear terahertz spectroscopy. Moreover, it is a compelling demonstration of spatio-temporal control of optical pump fields for plasma generation/emission, possibly of interest also to groups generating higher-frequency emission. That being said, I anticipate that the complexity of the setup and custom optical components will limit how broadly this approach is adopted.
In any case, I recommend acceptance for publication without any further changes.

Open Access This Peer Review File is licensed under a Creative Commons Attribution 4.0 International License, which permits use, sharing, adaptation, distribution and reproduction in any medium or format, as long as you give appropriate credit to the original author(s) and the source, provide a link to the Creative Commons license, and indicate if changes were made.
In cases where reviewers are anonymous, credit should be given to 'Anonymous Referee' and the source.
The images or other third party material in this Peer Review File are included in the article's Creative Commons license, unless indicated otherwise in a credit line to the material. If material is not included in the article's Creative Commons license and your intended use is not permitted by statutory regulation or exceeds the permitted use, you will need to obtain permission directly from the copyright holder.
To view a copy of this license, visit https://creativecommons.org/licenses/by/4.0/

Dr. Jeremy J. Pigeon
Staff Scientist and Group Leader
Ultrafast Plasma Photonics Group
Plasma & Ultrafast Science and Engineering
jepi@lle.rochester.edu

May 11th, 2026

We are grateful to the referees for their expert advice which has resulted in a significantly improved manuscript. Below, we respond to the referees' individual points. For clarity, the reviewer comments appear in italics. Our general responses are in normal black font and changes to the manuscript appear in blue.

On behalf of all authors,
Jeremy J. Pigeon

Reviewer 1:

1. *One significant advantage of the ultrashort flying focus technique is the ability to produce a flying focus with a velocity variation of 0.3%, all while keeping the pulse duration below 100 fs. Could you estimate or measure the effective pulse duration of your initial 50 fs laser pulse at the focal point of your parabola? The THz yield is sensitive to the initial pulse duration; therefore, this parameter is important for improving the THz conversion.*

We agree with the referee that this is an important point that should have been discussed in more detail in the manuscript. To estimate the temporal pulse broadening induced by the flying focus optics we have performed separate spectral interferometric measurements of both the trajectory and the impulse frequency response of the echelons and axiparabola used for this experiment. These measurements were detailed in reference [10], wherein we first demonstrated the ultrashort flying focus using the axiparabola and echelon pair. In reference [10], the flying focus optics broadened an equivalent 15 fs pulse to 30 fs yielding an impulse response function of approximately 26 fs (all FWHM). Using this impulse response, we can calculate that the 50-fs pulse, as measured with a commercial FROG device, was broadened to approximately 56 fs FWHM. Although we couldn't measure this directly, we believe that the reasonable optical-to-THz conversion efficiency indirectly confirms this result.

We have modified both the main text on page 4 and the supplemental material on page 10 to address this point in detail. In the main text:

Note that transmission losses of the telescope and slight temporal broadening from the flying focus optics resulted in an approximately 1.5-mJ, 56-fs FWHM laser pulse in the focal range of the axiparabola (see Methods).

In the methods section titled **Production of an ultrashort flying focus**

The spectral interferometric measurements were also used to measure the impulse frequency response of the echelon and axiparabola system, which was found to be approximately 26 fs FWHM [10]. Using this impulse frequency response, we estimate the pulse duration to be 56 fs FWHM in the focal range of the axiparabola.

2. *In the case of the superluminal ionization front, a conical-shaped THz radiation is observed, which is attributed to Cerenkov-like emission. A similar observation was made in reference [9], where the authors predicted an increase in THz energy for superluminal velocities compared to conventional focusing and a decrease in THz energy for subluminal velocities. Their velocities were much higher, but do your experiments or simulations show similar trends?*

We thank the referee for asking this question. We did observe a 1.4× increase in the conversion efficiency for the superluminal flying-focus trajectory relative to the subluminal case but neglected to mention this in the original manuscript. As the referee points out, a similar trend was previously reported in reference [9] and also predicted via the simulations presented in [7]. We have revised page 6 of the manuscript to mention this:

Note that the superluminal flying focus also resulted in slightly higher (1.2×10^{-4}) conversion efficiency as compared to the subluminal case (0.86×10^{-4}). A similar trend was previously reported in [9] and predicted by simulations [7].

In responding to the referees, we also thought it would be useful to directly state the measured conversion efficiencies for both flying focus velocities and the conventional focusing geometry. This appears on pg. 4 of the revised manuscript:

The transmission losses of the telescope and slight temporal broadening from the flying focus optics resulted in an approximately 1.5-mJ, 56-fs FWHM laser pulse in the focal region of the axiparabola (see Methods). For comparison, THz was also produced using a 3.0-mJ, 50-fs FWHM pulse with a conventional focusing geometry that produced a comparable filament length as the flying focus (see Methods). The conventional geometry, subluminal flying focus, and superluminal flying focus produced THz conversion efficiencies of 0.84×10^{-4}, 0.86×10^{-4}, and 1.2×10^{-4}, respectively.

3. *I am impressed by the section on characterizing the THz focusability. What is the proportion of THz energy in the spectral range above 10 THz? Based on the EO sampling measurement, it looks like this high frequency part is negligible and that a large part of the signal measured by the HF bolometer is actually at low frequency (unless you use a low pass filter).*

We thank the referee for their positive comment and interesting question. In truth, we consider that the spectral content in the flying focus driven THz source is still an open question and that EOS measurements can be somewhat misleading. The effect of phase matching in the 100 μm thick GaP crystal and the finite pulse duration used as the probe limits the frequencies that EOS can detect resulting in the preferential detection of frequencies from 1 – 7 THz (*e.g.* Leitenstorfer, *et. al.*, APL **74**(11) 1999). Although two-color THz sources tend to have most of their spectral energy density below 10 THz, it is generally accepted that the two-color THz source produces a supercontinuum with frequency content much greater than that measured by electo-optic sampling. We are currently pursuing complementary measurements using Fourier transform infrared spectroscopy (FTIR) that should give us a more accurate measurement of the true frequency content. Nevertheless, from our previous work on the simulation of THz production using a flying focus we estimate that, for the subluminal trajectory, ~ 75% of the THz energy lies below 10 THz. In the case of the superluminal trajectory, however, we estimate that only 40% of the THz energy lies below 10 THz.

The spatial measurements are complicated by this extremely broadband THz emission. Although most of the THz energy may be < 10 THz, the frequency dependent THz spot size results in a much tighter focus at

higher frequencies. As a result, the measurements near focus tend to be dominated by the highest frequencies that the microbolometer can detect, causing the focus measured by the HF microbolometer array to be near 30 THz as estimated from the diffraction limited spot size. For the LF microbolometer array the focus is dominated by ~ 4 THz which is, again, at the highest range of frequencies detected by this array. Despite these subtleties in our spatial measurements, we are confident that the flying focus (both sub- and superluminal trajectories) produces a tighter focal spot than the conventional case since we have performed these measurements under otherwise identical conditions.

4. *Figure 2 shows that the numerical simulations qualitatively reproduce the shape of the measured THz profile, but the agreement is not very good. These simulations are very complex, but one important aspect is unclear in your description of two-color THz generation: The photocurrent created by the two-color pulse depends strongly on the phase difference between the 800-nm pulse and the 400-nm pulse. However, due to dispersion, these two pulses have different velocities in air, which induces phase slippage during propagation and eventually leads to destructive interference in the THz emission when the plasma source is too long. This is why most people limit the length of the plasma source with a two-color laser to a few millimeters. In your case, however, you produce a relatively long plasma source (6 mm), yet you do not discuss the problem of phase slippage between the 800- and 400-nm pulses. You only mention the ionization front velocity. However, to achieve optimal THz generation, it appears that you must independently control the 800-nm and 400-nm pulses to counteract the dispersion in air and plasma. Could you comment on this?*

We thank the referee for this comment and agree that the control over the relative phase between the fundamental and second-harmonic pulse should be explained in more detail. The referee is mistaken, however, that the coherence length is only a few millimeters. In air, the coherence length defined as $L_d = \lambda/2[n(\omega) - n(2\omega)]^{-1}$ is approximately 22 mm (See reference [36] and others). As a result, the THz generation in this experiment was within one coherence length. In both experiment and the simulation, the relative phase was optimized to maximize the THz yield. In the experiment, this was accomplished by rotating the 1-mm thick MgF_2 plate (see Methods). Similarly, in our simulations multiple runs were used to determine the optimal relative phase of the fundamental and second harmonic such that the THz conversion was maximized. We have included these details in both the main manuscript and Methods sections.

In the main manuscript on pg. 4:

Finally, a dichroic waveplate provided parallel polarization between the harmonics and the relative phase was controlled by rotating a 1-mm thick MgF_2 plate that enabled THz power conversion efficiencies on the order of 10^{-4} in air.

In the Methods section titled **Simulations of THz production:**

To maximize THz yield multiple trial runs were employed to empirically determine the optimal phase between the fundamental and second-harmonic pulse.

5. *Figure 4 shows the THz temporal shape and the corresponding spectra measured with the two flying focus cases. Could you also compare these with the "conventional case"? Also, the measured signal appears quite noisy for such a high-energy THz pulse. Could you comment on the origin of the numerous absorption lines in Fig. 4b's spectrum? Are they due to the GaP crystal*

or humidity in the air?

We thank the referee for this question and agree that the modulations on the THz spectrum should be explained and compared to the conventional focusing geometry. As the referee suggested, the modulations present are from water absorption in laboratory air and temporal reflections in the electro-optic crystal. To avoid cluttering Fig. 4 in the main manuscript, we have included a new figure in the methods section (Fig. S2) that directly compares the temporal profile and spectral content with the conventional focusing geometry performed under identical conditions (100 μm thick GaP and in laboratory air) to that obtained with the subluminal flying focus. In doing so, we have also performed a time-domain filter on all the EOS traces prior to taking a Fourier transform so that each spectrum can be compared without the additional modulation.

We have modified pg. 5 of the main manuscript to discuss this:

The peak fluence of the THz radiation was also determined from these measurements [see inset of Fig. 3(b)], which show that the flying focus produces higher intensity HF radiation than the conventional focusing geometry. Moreover, measurements of the LF THz beam show an increased fluence obtained with the subluminal velocity indicating that this velocity produced more LF radiation than the superluminal case.

We have also included a new supplemental figure and surrounding text in the Methods section "**Two-color THz production with conventional focusing geometry**" :

In addition to the spatial beam profile measurements presented in this article, the electric field of the THz produced with the conventional focusing geometry was measured at its focus. Electro-optic sampling measurements under identical conditions were performed and the resulting Fourier transform is presented in Fig. S2. For comparison, this data is presented with that obtained with each flying focus velocity [data presented in Fig. 4(b)]. In Fig. S2, a temporal filter was applied after the main THz pulse (for times greater than 1 ps) prior to performing the Fourier transform to reduce the spectral modulations attributed to H_2O absorption in laboratory air and temporal reflections in the electo-optic crystal.

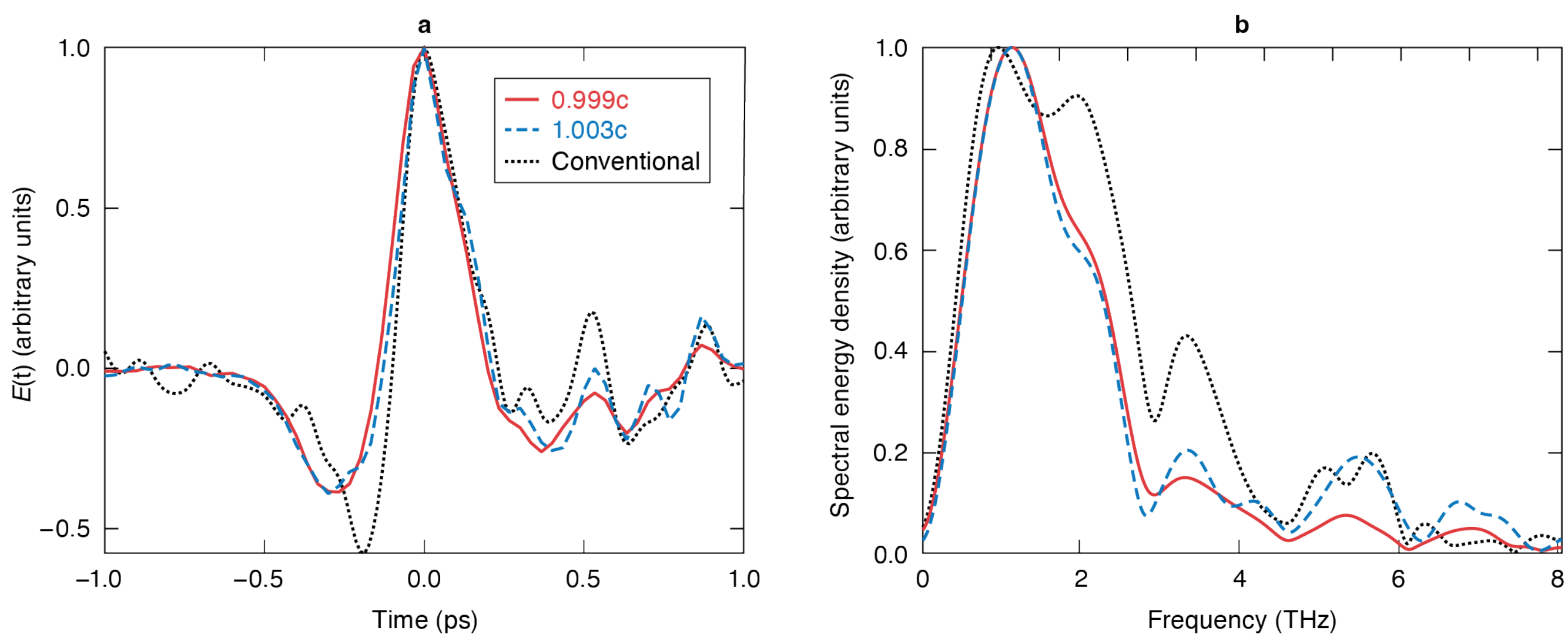

Figure S2: (a) The normalized electric field of THz produced with the conventional focusing geometry and each flying-focus pulse both obtained via electro-optic sampling in 100-μm thick GaP crystal. (b) The associated spectra obtained via Fourier transformation after application of a temporal filter beyond 1 ps to reduce the spectral modulations present in Fig. 3(b).

<u>Reviewer 2:</u>

1. *However, a central claim of the work, the spectral/temporal control of the terahertz radiation is not well supported by the provided data, as only two different data sets (1 subluminal versus 1 superluminal) are shown.*

We agree with the referee that additional echelons able to provide a larger range of velocities would be beneficial. Unfortunately, the production of these complex echelons requires a significant investment of time and resources to fabricate, perform metrology, and coat by an outside vendor. In addition to the fabrication and metrology, detailed spectral interferometric measurements such as those detailed in reference [10] were also applied to ensure that the focal velocity and temporal pulse duration were correct prior to their application for this proof-of-principle study. Now that the potential of this technology is clear, future work will be devoted to producing a larger range of focal velocities using either the echelons described in this study or a new technique that relies on a spatial light modulator and deformable mirror to produce a rapidly programmable flying focus pulse [48]. This qualifier aside, we are confident that the flying focus can alter the spectral properties of the emitted THz since the experimental measurements are in qualitative agreement with numerical simulations. To add extra weight to this aspect of the study, we have performed additional analysis of the peak fluence obtained with both the high-frequency (HF) and low-frequency (LF) microbolometer arrays that is presented as an inset in Fig. 3b. Although the conversion efficiency is 1.4× higher with the superluminal velocity than the subluminal, the peak fluence measured with the LF microbolometer array detecting 0.4 – 4 THz radiation was 4× larger with the subluminal flying focus suggesting that the subluminal velocity produces much more low frequency radiation. Moreover, we have also measured a 2× larger electric field via electo-optic sampling (EOS) under identical conditions for THz produced with a subluminal velocity. This measurement is consistent with the increased fluence measured with the LF micrbolometer array since EOS in 100 µm thick GaP is only sensitive to frequencies below 7 THz.

We have modified pg. 5 of the main manuscript to discuss the increased fluence at low frequencies produced with the subluminal flying focus:

The peak fluence of the THz radiation was also determined from these measurements [see inset of Fig. 3(b)], which show that the flying focus produces higher intensity HF radiation than the conventional focusing geometry. Moreover, measurements of the LF THz beam show an increased fluence obtained with the subluminal velocity indicating that this velocity produced more LF radiation than the superluminal case.

And have explicitly stated the THz conversion efficiencies on pg. 4 of the revised manuscript:

Note that transmission losses of the telescope and slight temporal broadening from the flying focus optics resulted in an approximately 1.5-mJ, 56-fs FWHM laser pulse in the focal range of the axiparabola (see Methods). For comparison, THz was also produced using a 3.0-mJ, 50-fs FWHM pulse with a conventional focusing geometry that produced a comparable filament length as the flying focus (see Methods). The conventional geometry, subluminal flying focus, and superluminal flying focus produced THz conversion efficiencies of 0.84×10^{-4}, 0.86×10^{-4}, and 1.2×10^{-4}, respectively.

Finally, we have also changed our presentation of electro-optic sampling measurements to normalize the electric field to the subluminal case as opposed to each measurement being normalized to itself. We have also improved our electo-optic sampling analysis to include an average of 5 traces taken near the best focus to attach an error bar to the spectra. The revised Fig. 4 and accompanying text are on pgs. 5-6:

The electric field of the THz produced with the subluminal flying focus was measured to be ~ 2× larger than the superluminal case. This observation was consistent with the increased fluence at low frequencies determined by the LF microbolometer array [see inset of Fig.4(b)] since the electo-optic sampling method is only sensitive to frequencies below 7 THz. Regardless of longitudinal position about the focus, the superluminal flying-focus produced higher frequency THz radiation than the subluminal velocity [Fig. 4(b)]. To illustrate this, both the time- and frequency-domains are presented as the mean and standard deviation of 5 measurements taken near the THz focus (z = 0). With the subluminal flying focus, the ionization front lags the THz pulse and generates new radiation behind that generated upstream thereby lengthening the pulse in the time domain and lowering the THz frequency. The ionization front driven by the superluminal flying focus, however, creates new THz radiation ahead of the upstream radiation that interferes in a Cherenkov-like shock that shortens the THz pulse duration generating higher frequencies than the subluminal case. The modulations present in Fig. 4b are likely due to H_2O absorption in the laboratory air and temporal reflections in the electro-optic crystal. Figure S2 in Methods shows this data after time-domain filtration to remove the modulations behind the main THz pulse along with electo-optic sampling data obtained using the conventional focusing geometry.

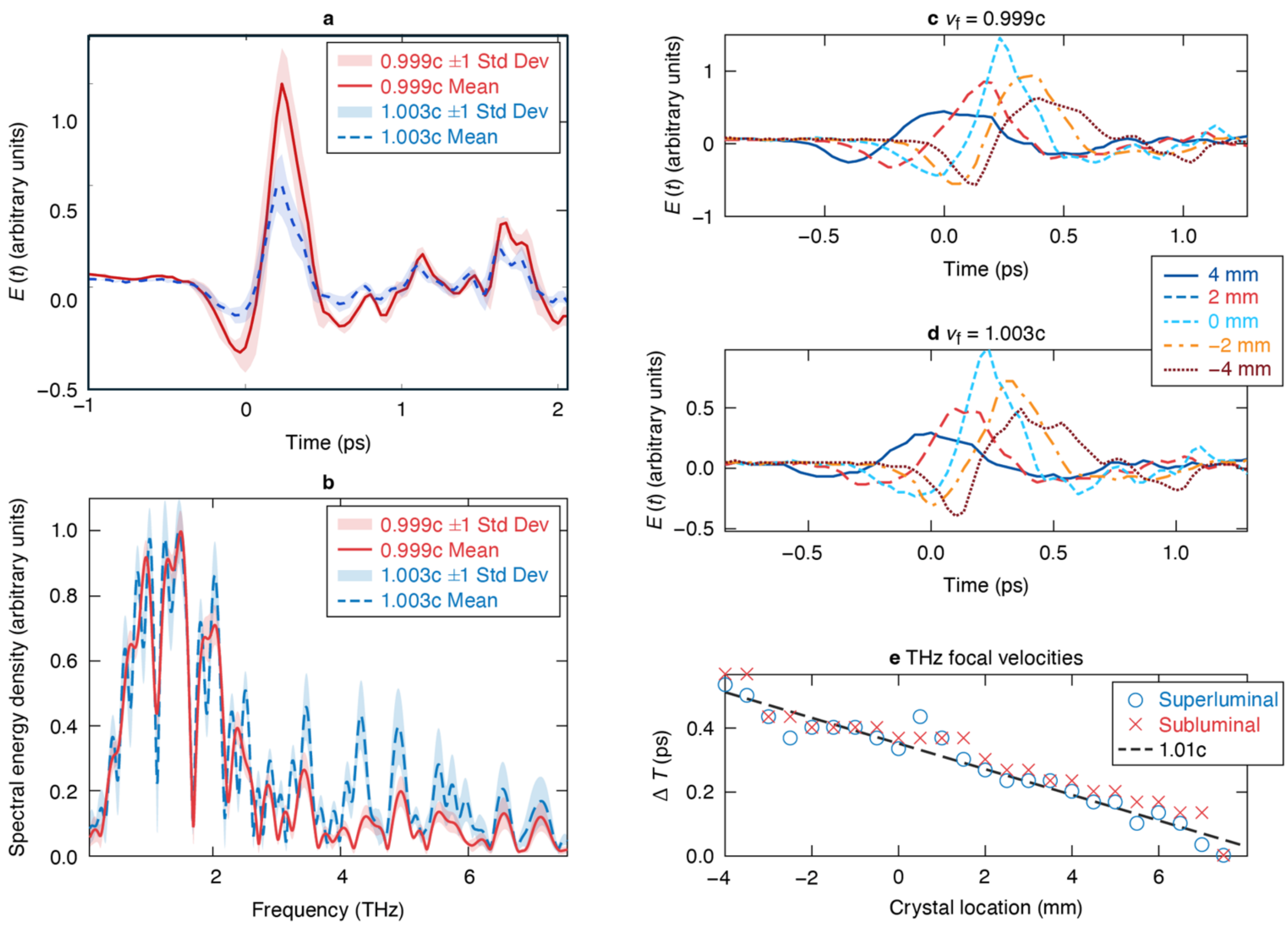

Figure 4: The focal velocity of the two-color flying focus pulse can be used to tune the spectrum but does not affect the focal velocity of the THz radiation. (a) The mean THz electric field and standard deviation from 5 measurements performed using electro-optic sampling near the THz focus at z = 0 (see Methods) for the sub- and superluminal flying-focus. (b) The Fourier transform of the mean and +/- one standard deviation of the data presented in (a). Time-domain measurements of THz produced with the (c) subluminal and (d) superluminal flying-focus pulses, measured about the focus of the second OAP in Fig. 1. (e)

Measurements of the focal velocity of the THz pulse obtained using electro-optic sampling by translating the crystal through the THz focus.

2. *The content of Fig. 4e is not properly explained though. What is shown here? What is Delta T? The plot title is also wrong.*

We thank the referee for this comment and agree that Fig. 4(e) should be explained in more detail. Figure 4(e) depicts measurements of the THz focal velocity obtained via electo-optic sampling in a 100 μm thick GaP crystal following the procedure laid out in reference [46]. Essentially, we have performed pump-probe measurements for various crystal locations within the focus of the THz pulse and have observed how the peak of the time-domain trace changes as a function of that crystal location. Here, ΔT is the arrival time of the peak of the THz electric field in the electo-optic sampling measurement. If the THz pulse was propagating at the same group velocity of the near-IR probe pulse, then this would be a horizontal line (*i.e.* the probe and the THz pulse would be co-timed regardless of the crystal position in the THz focal geometry). The purpose of this measurement was to investigate whether the flying focus velocity had any effect on the focal velocity of the THz pulse, and we found that it did not. Moreover, the results were consistent with other measurements [46]. We have revised the text surrounding Fig. 4 and the title of Fig. 4e to clarify this:

To determine the focal trajectory of the THz, a series of electro-optic sampling measurements for the sub- and superluminal flying-focus were obtained by translating the electo-optic crystal through the THz focal region and observing the time at which the THz and near-IR probe were synchronized. These measurements show that the peak THz field moves in time relative to the group velocity of the probe pulse (ΔT) and that this time depends on the focal location [Fig. 4(c-d)]. When plotting these measurements as a function of the crystal location, the THz radiation was found to propagate with a focal velocity near 1.01c, regardless of the flying-focus velocity used to produce the THz [Fig. 4(e)].

3. *The presented work is clearly focused on the experimental realization of previous theoretical work (Refs. 7,8). In this regard, the manuscript often fails to provide the relevant physical background to the reader. Without knowing the referenced theory work in depth, it is not clear why a flying focus should enable tighter THz beam focusing than conventional two-color photoionization (line 152).*

We are grateful to the referee for this comment and agree that a more detailed discussion of the relevant physics would be useful. In our response, we have also discovered a typographical error in Eq. (1) that is now corrected (the ratio of the THz phase velocity to the ionization front velocity was wrongly reciprocated). We have significantly modified the introduction on Pg. 2 to address this and to hopefully provide a satisfactory physical picture:

Under conventional focusing, however, THz radiation constructively interferes in a Cherenkov-like cone with an angle ϕ determined by the velocity of the ionization front v_f and the phase velocity of the THz v_{THz} [44, 45]:

$$\phi = \cos^{-1}(v_{THz}/v_f) \qquad (1)$$

The velocity of the ionization front is faster than the THz phase velocity due to external focusing increasing the intensity resulting in photoionization occurring earlier in the two-color pulse as it comes to focus. More generally, the emission angle depends on the beam geometry, linear dispersion, and nonlinear propagation of the two-color pulse making it difficult to predict or control in an experiment where parameters such as

gas pressure are varied to optimize THz conversion efficiency. Moreover, in the filamentary propagation that typically accompanies THz generation, cycles of focusing and de-focusing can cause THz to be emitted in a range of Cherenkov-like emission angles resulting in a larger effective source size that limits how tightly the THz can be focused for applications.

We also agree with the referee that the improved focus of the THz produced via the flying focus ought to be explained in detail and hope that revisions to the introduction detailed above and the following modifications to the results section on Pg. 5 clarify this:

Measurements of the improved focusing of the flying-focus-produced THz are consistent with previously simulated THz virtual source sizes [7] and can be understood by considering the angle of emission (see Eq. 1). As opposed to the conventional geometry that produces a range of Cherenkov-like angles due to nonlinear propagation, the flying focus produces THz at a well-defined angle due to the constant ionization front velocity resulting in improved focusing.

4. *In addition, it is not clear why a superluminal flying-focus produces higher frequency content than the subluminal (line 171).*

We agree that this should be explained in more detail and have modified the results section pgs. 5-6 to explain why the frequency content depends on the focal velocity:

With the subluminal velocity flying focus, the ionization front lags the THz pulse and generates new radiation behind that generated previously thereby lengthening the pulse in the time domain and lowering the THz frequency. The ionization front driven by the superluminal trajectory, however, creates new THz radiation ahead of the previously generated radiation that interferes in a Cherenkov-like shock that shortens the THz pulse duration generating higher frequencies than the subluminal case.

5. *As mentioned above, additional EOS data in Fig. 4 for more focal velocities would strengthen the work and the claim of spectral control.*

We thank the referee for this critical feedback. Please see our response to comment #1 above.

6. *The agreement between experiment and simulation in Fig. 2 is debatable. There is no length scale provided in the simulated data. A more detailed discussion of agreements and deviations is desirable.*

While we agree with the referee that we were unable to obtain exact quantitative agreement between the experiment and simulation, this is not unusual for experiments involving laser filamentation. Moreover, we believe that the features of the spatial beam profile in experiment and simulation are in good qualitative agreement since the subluminal velocity flying focus generates a fluence profile that is peaked on-axis while the superluminal flying focus creates a ring-shaped beam. We are grateful to the referee for pointing out that the spatial scale was missing from our simulation data. We have now included this spatial scale in the revised Fig. 2. Note that the experimental beam profiles were measured at a distance of 5 mm from the focus of the OAP while the simulation data was measured 100 mm from the filament. When accounting for the divergence of the beam and the 0.44× magnification expected from the ratio of the focal lengths of the two OAPs used in the experiment, the beam size and divergence in both experiment and simulation are in good agreement. Nevertheless, we agree with the referee that a more detailed discussion of agreements and deviations would be useful.

The remaining discrepancies occur mainly because of experimental realities including aberrations in the optics, details of the initial pulse profile and spatio-spectral phase, as well as the introduction of a tri-foil shadow due to our up-collimating telescope. The simulations also did not account for absorption and dispersion of IR-active minor air constituents such as H_2O vapor which could play a role. Finally, due to computing constraints, the UPPE simulations required filtering of THz frequencies below 1 THz to save computational time. Our microbolometer arrays, especially the low-frequency array, is sensitive to these frequencies which is why we see the largest disagreement with these spatial measurements [i.e. Fig. 2(c) Fig. 2(e) as compared to Fig. 2(g) and Fig. 2(h)]. We are currently working on a combination echelon and axiparabola that will simplify the flying focus implementation and hopefully provide a more quantitative match with improved simulation capabilities.

We have modified the results section on pg. 4 to address the some of these points:

Other discrepancies are mainly related to experimental realities including aberrations in the optics, details of the initial pulse profile and spatio-spectral phase, as well as the introduction of a tri-foil shadow due to the up collimating telescope. The simulations also did not account for absorption and dispersion of IR-active minor air constituents such as H_2O vapor.

We have also modified the caption of Fig. 2 to clarify both the simulated and experimental beam profiles:

Figure 2: Sub and superluminal two-color flying focus pulses produce on-axis and conical emission, respectively. THz far-field profiles produced with (a), (c), subluminal and (b), (d) superluminal flying-focus trajectories measured with a (a), (b) high frequency (HF) and (c), (d) low-frequency (LF) microbolometer arrays. Panels (e), (f), (g), and (h) show THz emission profiles from simulations initialized with corresponding focal velocity and frequency range of the measurements above them. Note that the experimental beam profiles were measured 5 mm away from the OAP focus and that the simulations were measured 100 mm from the filament. The spatial sizes were in agreement after accounting for the differences in magnification.

For completeness, we have also included the focal lengths of the OAPs at the end of the Methods section **THz spatial beam profile measurements:**

The THz radiation was re-collimated by an f/3 (228.6 mm focal length) off-axis parabolic mirror (OAP) before being focused by a second f/2 (101.6 mm focal length) OAP.

7. *The structure of the paper could be optimized to avoid repetitions. Some results are presented already before the beginning of the Results section (line 99 and subsequent).*

We agree with the referee that these lines are redundant and have removed these lines from the revised manuscript.

8. *Minor language issues: doubling of words in l. 383, "MgF2 plate" (l. 408), "Wallaston prism" (l. 422), missing word? in l. 110, "mission" (l. 143)*

We thank the referee for pointing out these errors and have revised the manuscript accordingly.

Reviewer 3:

1. *As with any modified experimental scheme, requiring a more complex apparatus, one needs to clearly address any disadvantages that might arise, not only the apparent benefits over the "conventional" approach.*

We agree with the referee that the difficulty in producing the flying focus should be discussed in detail. We initially included some limitations in the discussion section on pg. 7 but have added to this in response to the referee's comment. The revised discussion section:

This technique provides control over the spatial and temporal properties of THz radiation but has important limitations. Most notably, precise alignment of the echelon and axiparabola was required to produce the correct focal trajectory of the flying focus. With increasing misalignment, the THz profile began to resemble the profiles produced using the axiparabola alone as the echelon was no longer properly structuring the radial group delay of the pulse. In addition, spatiotemporal structuring with large aperture optics requires a reflective telescope and multi-meter propagation that necessitates a time-delay compensator for nonlinear frequency conversion with a collimated beam. Finally, the use of an all-reflective telescope with center obstruction, resulted in a 50% reduction in the pulse energy (see Methods).

2. *The authors should address better the true gain from the subluminal case compared to conventional. In the Introduction, the conventional conical emission is described only as a disadvantage ("impossible to control", "complicates collection ... of the radiation"). In practice, the conventional cone angle is still very manageable, the beam is linearly polarized, and subsequent foci still have a clean on-axis maximum (the cone only leading to weak side lobes). Moreover, the "donut" collimated beam even has advantages in terms of allowing on-axis elements to filter the optical pump beams and reintroduce collinear optical beams for detection. If the authors can demonstrate that there is a true efficiency gain from the FF geometry, this needs to be stated (although I suspect this is not the case, as it is not addressed in the manuscript).*

We agree with the referee that the donut-shaped beam produced in a conventional set-up is not inherently bad and has some benefits. Indeed, THz generation with the flying focus can also produce such a beam and this may be advantageous to some applications as the referee points out. We have modified the introduction section to soften some of this language and to add additional physical insight.

The velocity of the ionization front is faster than the THz phase velocity due to external focusing increasing the intensity resulting in photoionization occurring earlier in the two-color pulse as it comes to focus. More generally, the emission angle depends on the beam geometry, linear dispersion, and nonlinear propagation of the two-color pulse making it difficult to predict or control in an experiment where parameters such as gas pressure are varied to optimize THz conversion efficiency. Moreover, in the filamentary propagation that typically accompanies THz generation, cycles of focusing and de-focusing can cause THz to be emitted in a range of Cherenkov-like emission angles resulting in a larger effective source size that limits how tightly the THz can be focused for applications.

We also thank the referee for their comment on the efficiency gain possible with the flying focus. Although we did not discuss this in the initial manuscript, the superluminal echelon did produce a slightly higher conversion efficiency (1.2×10^{-4}) than that obtained with the conventional focus (0.84×10^{-4}). The subluminal produced a very comparable conversion efficiency (0.86×10^{-4}) as that produced with the conventional focusing. Although the increase in conversion efficiency was slight, the production of the flying focus resulted in a 50% reduction in pulse energy (1.5 mJ) from what was used to generate THz with conventional focusing (3.0 mJ). This loss in pulse energy was due to up-collimation of the input beam with an all-

reflective telescope with a center obstruction that diminished the total energy of the beam (see Methods). Although somewhat speculative, increasing the pulse energy and optimizing the flying focus velocity may eventually enable better conversion efficiency than conventional focusing. To address this point, we have modified the results section of the manuscript to explicitly state the measured conversion efficiencies:

For comparison, THz was also produced using a 3.0-mJ, 50-fs FWHM pulse with a conventional focusing geometry that produced a comparable filament length as the flying focus. The conventional geometry, subluminal flying focus, and superluminal flying focus produced THz conversion efficiencies of 0.84×10^{-4}, 0.86×10^{-4}, and 1.2×10^{-4}, respectively.

3. *The apparent increase in spectral intensity (from ~3-7 THz) in Fig. 4(b) for the superluminal case is not convincing. Why are the spectra strongly modulated - due to reflections in the time-domain? The Fourier analysis should be improved, including error margins for the high-frequency signal to ensure this section of the spectrum is reliable. As mentioned in point 1 above, the spectrum from the conventional method has to be included.*

We agree with the referee that the increase in the high frequency region in the spectra is slight [Fig. 4(b)]. Our previous simulation paper [Ref. 7] predicted that the superluminal flying focus would produce higher frequency radiation but this change is most pronounced for frequencies > 10 THz that we were unable to detect via electo-optic sampling in 100 μm thick GaP. That said, we have consistently observed that the subluminal flying focus produces an ~ 2× larger electric field as determined via electro-optic sampling measurments under identical conditions. Since these measurements are only sensitive to relatively low frequency radiation, this strongly suggests that the subluminal flying focus produces more low frequency radiation than the superluminal case. We have changed the presentation of the data in Fig. 4(a) to normalize the electric field to that obtained in the subluminal case as opposed to normalizing each electric field to itself to show this. To associate error bars to the spectral measurements, we have also performed additional analysis to average 5 time-domain and spectral measurements at points close to the focus. The revised Fig. 4 now shows the average value along with shaded bands that depict +/- one standard deviation from this average. As can be seen in the revised Fig. 4 (see below), the results of this analysis do not change our main conclusion. We are grateful to the referee for this comment, that resulted in an improved presentation of these results. In response to the referee's comment #5 below, we have also analyzed the peak fluence measured with each flying focus velocity for each microbolometer array. These results clearly indicate that the subluminal velocity creates more low-frequency content as the measured fluence on the low-frequency microbolometer array was ~ 4× larger than the that produced with the superluminal velocity flying focus. This is despite the fact that the superluminal flying focus is ~ 1.4× more efficient overall and is consistent with the 2× larger electric field measured via electo-optic sampling.

We have also included the electo-optic sampling trace and associated Fourier transform obtained with the conventional focusing geometry plotted with those obtained with each flying focus velocity. We decided to present this as a new supplemental Fig. S2 in the Methods section of the paper. In doing so, we have presented data only obtained at the position of best THz focus (z = 0 in Fig. 3 and Fig. 4) and have also filtered the EOS trace in the time-domain prior to performing the Fourier transform. We believe the modulations present in Fig. 4 are from water absorption in laboratory air and temporal reflections in the GaP crystal which were removed in Fig. S2 by time-domain filtration. We have decided to leave the modulations present in Fig. 4, however, since this is a more accurate description of what was directly measured in the experiment.

The revised Fig. 4 and surround text on pgs. 5-6 :

The electric field of the THz produced with the subluminal flying focus was measured to be 2× larger than the superluminal case. This observation was consistent with the increased fluence at low frequencies determined by the LF microbolometer array [see inset of Fig.4(b)] since the electo-optic sampling method was only sensitive to frequencies below 7 THz. Regardless of longitudinal position about the focus, the superluminal flying-focus produced higher frequency THz radiation than the subluminal velocity [Fig. 4(b)]. To illustrate this, both the time- and frequency-domains are presented as the mean and standard deviation of 5 measurements taken near the THz focus ($z = 0$). With the subluminal flying focus, the ionization front lags the THz pulse and generates new radiation behind that generated previously thereby lengthening the pulse in the time domain and lowering the THz frequency. The ionization front driven by the superluminal flying focus, however, creates new THz radiation ahead of the previously generated radiation that interferes in a Cherenkov-like shock that shortens the THz pulse duration generating higher frequencies than the subluminal case. The modulations present in Fig. 4b are likely due to H_2O absorption in the laboratory air and temporal reflections in the electro-optic crystal. Figure S2 in Methods shows this data after time-domain filtration to remove the modulations behind the main THz pulse along with electo-optic sampling data obtained using the conventional focusing geometry.

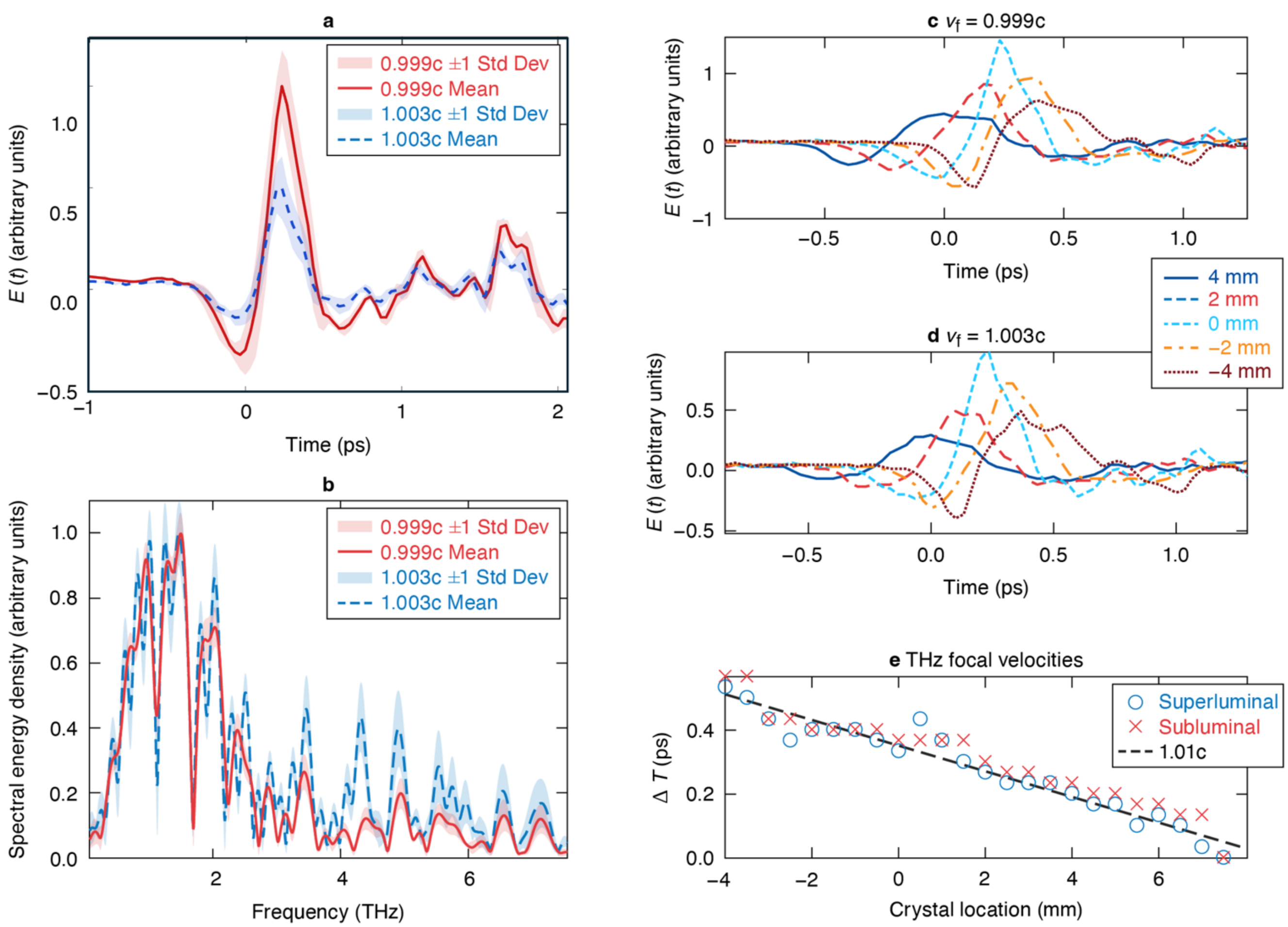

Figure 4: The focal velocity of the two-color flying focus pulse can be used to tune the spectrum but does not affect the focal velocity of the THz radiation. (a) The mean THz electric field and standard deviation averaged from 5 measurements performed using electro-optic sampling near the THz focus at $z = 0$ (see Methods) for the sub- and superluminal flying-focus velocities. (b) The Fourier transform of the mean and +/- one standard deviation of the data presented in (a). Time-domain measurements of THz produced with the (c) subluminal-velocity and (d) superluminal-velocity flying-focus pulses, measured about the focus of

the second OAP in Fig. 1. (e) Measurements of the focal velocity of the THz pulse obtained using electro-optic sampling by translating the crystal through the THz focus.

4. *One important issue is that by spreading the focal region with a FF, the peak intensities reached are lower. Hence, plasma densities will be lower, which will be a penalty unless one reaches the saturation regime. The authors need to specify what conditions (i.e. pump opulse energies) the FF could maintain the THz generation efficiency.*

We agree that we should have estimated the peak intensity achieved in this experiment. Due to the f/5 focusing geometry of the axiparabola, the peak intensity used in this experiment is on order 10^{14} W/cm^2 and comparable to a conventional experiment. This discussion can now be found in the revised Methods section titled "Production of an ultrashort flying focus":

> The peak intensity can be estimated by calculating the peak intensity and reducing it by the ratio of the Rayleigh length to the extended focus which is ~ 1/237 for the experimental conditions. As a result, we estimate a peak intensity of ~5.5×10^{14} W/cm^2.

5. *The used definition of the beam radius is not rigorous. Here one should use the rms width calculated relative to the center of mass, and also the on-axis peak fluence. At present, one of the most important conclusions is based on this subjective metric.*

We are grateful to the referee for critiquing our beam analysis. We have followed your suggestion and have re-analyzed our beam profile data using the second moment of the fluence relative to its centroid (*i.e.* the first moment). The revised Fig. 3 now includes the results of this analysis and includes both the x and y RMS width ($2\sigma_x$, $2\sigma_y$) for all three of the datasets presented in Fig. 3a. Our main conclusion, that the flying focus produces THz that can be focused to a ~ 2× smaller spot than that produced with the conventional geometry has not changed. However, the analysis shows that the RMS width of the subluminal velocity flying focus is reduced as compared to that produced with the superluminal velocity pulse (due to the absence of the lobes that are present by focusing a ring-shaped beam). We would also like to thank the referee for suggesting that we plot the peak fluence at the center of the beam for this data set. The peak fluence is now presented as an inset in the revised Fig. 3b. This analysis shows that both flying focus pulses produced a higher fluence at high frequencies than that produced with the conventional focusing geometry. Moreover, an analysis of the low frequency microbolometer array data shows that the subluminal flying focus produced ~ 4× higher fluence than the superluminal velocity suggesting that the THz frequency content produced with the subluminal velocity is lower frequency than that produced with the superluminal velocity. If not for the referee's advice, we would have overlooked this measurement that strengthens our claim that the flying focus velocity modifies the spectral content of the THz pulse.

We have modified the text on pg. 5 and Fig. 3 to present this improved data analysis:

Figure 3 demonstrates that the flying focus produced high-frequency THz radiation that was more focusable than that produced with a conventional focusing geometry. To directly compare the ring-shaped and on-axis beam shapes, the RMS beam width (σ_x, σ_y) was determined by calculating the second moment of the fluence with respect to two directions (x, y) measured relative to the beam centroid [46]. Figures 3(a) and 3(b) show the 2σ beam radii measured about the focus with the HF and LF microbolometer arrays, respectively. The sub- and superluminal flying focus produced THz focused to a minimum radius of 57 μm and 95 μm,

respectively, while the THz generated by conventional focusing had a minimum radius of 120 µm [Fig. 3(a)]. Measurements of the improved focusing of the flying-focus-produced THz are consistent with previously simulated THz virtual source sizes [7] and can be understood by considering the angle of emission (see Eq. 1). As opposed to the conventional geometry that produces a range of Cherenkov-like angles due to nonlinear propagation, the flying focus produces THz at a well-defined angle due to the constant ionization front velocity resulting in improved focusing. For the low-frequency measurements (0.4 – 4 THz), both flying-focus velocities produced THz that focused to a minimum spot size of ~ 360 µm, which corresponds to the diffraction limit. The peak fluence of the THz radiation was also determined from these measurements [see inset of Fig. 3(b)], which shows that the flying focus produces higher fluence HF radiation than the conventional focusing geometry. Moreover, measurements of the LF THz beam show an increased fluence obtained with the subluminal velocity indicating that this velocity produced more LF radiation than the superluminal case.

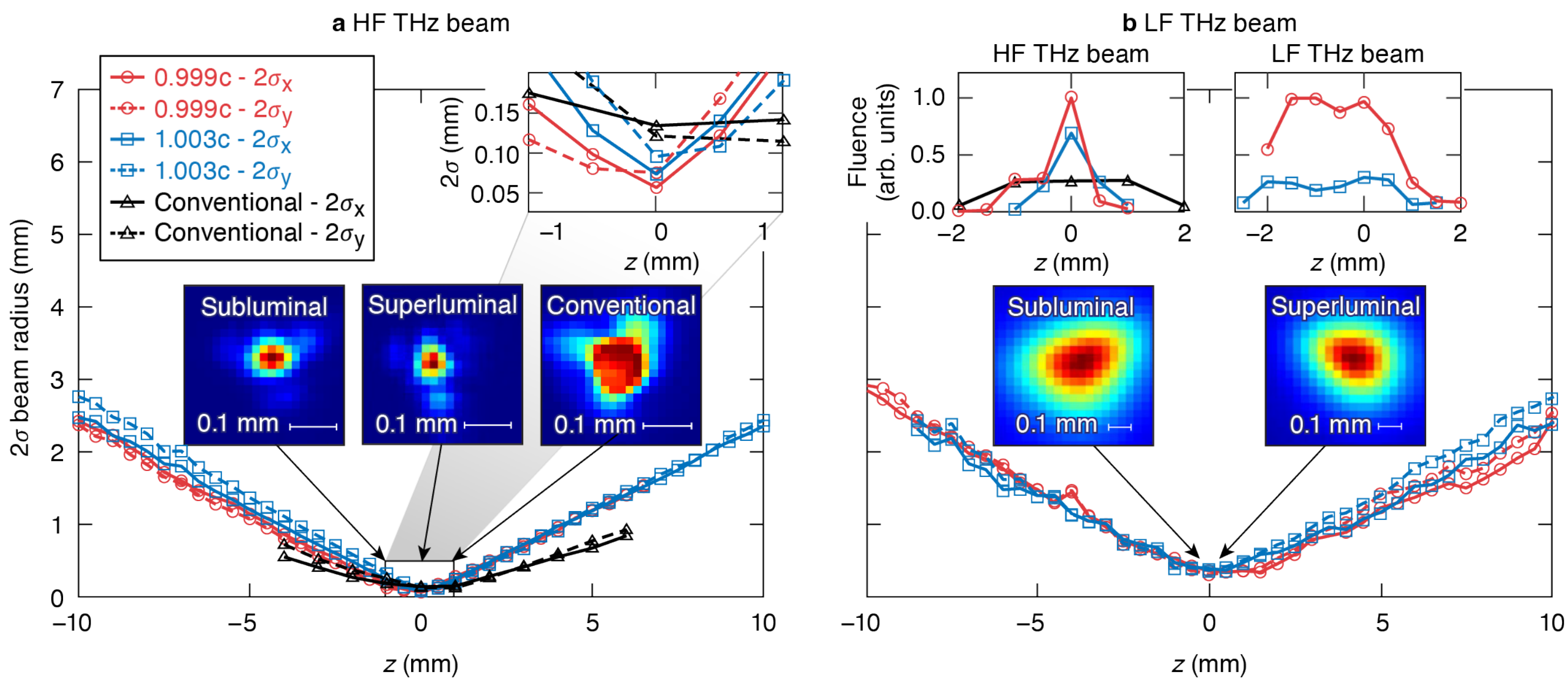

Figure 3: The THz radiation generated by the two-color flying focus pulse has better focusability than that produced by a conventional two-color pulse. (a) The RMS beam radius ($2\sigma_x$, $2\sigma_y$) and spatial beam profiles near focus of the THz beam measured with the HF microbolometer array and (b) LF microbolometer array.

6. *The beam profiles in Fig. 1(b,c) are initially confusing, and only become clear in reaching Fig, 2. The authors should state where they are measured relative to the focal plane (i.e. sufficiently far from the focus that they represent the shape of the collimated beam, not the focal profile).*

We agree with the referee and have modified the caption of Fig. 1 clearly state where these beam profiles were measured:

Figure 1: (a) Schematic representation of the experiment including formation of a two-color ultrafast flying focus using an axiparabola (AP) and echelon pair. (b) and (c) Visualization of the emitted wavefronts and experimental THz beam profiles generated by flying-focus pulses with (b) subluminal and (c) superluminal velocities. The beam profiles were measured 5 mm away from the focus and are representative of the shape of the collimated beam. DWP is a dichroic waveplate; OAP is an off-axis parabolic mirror; EOS is electro-optic sampling; HPFZ-Si is a high-purity float-zone silicon window.

7. Some minor issues: The Methods states a 4x magnification of the optical pump beams, but it is given as 10x in Fig. 1(a)? For the conventional method, was the beam expander also used? "Wallastan" -> "Wollaston".

We thank the referee for finding these errors and have corrected them in the revised manuscript. The beam expander produced a 4× magnification and was not used for the conventional focusing geometry. We have modified the Methods section: **Two-color THz production with conventional focusing geometry** to clarify this:

An initially 15-mm ($1/e^2$ diameter) beam was focused by a 500-mm-focal-length lens (f/33) to produce a filament of comparable length (6 mm) to that produced with the axiparabola and echelon configuration.
